# *Dachaihu* decoction ameliorates abnormal behavior by regulating gut microbiota in rats with propionic acid-induced autism

**DOI:** 10.3389/fmicb.2025.1535451

**Published:** 2025-02-13

**Authors:** Yangyang Zhang, Hang Li, Bolin Li, Yizhuang Li, Xuejun Chai, Sheng Li, Xia Xue, Honglei Li, Yonghong Zhao, Youcai Tang, Baoqi Yin, Pengju Zhao, Enyao Li, Pengya Feng

**Affiliations:** ^1^Department of Children Rehabilitation, Henan Key Laboratory of Rehabilitation Medicine, The Fifth Affiliated Hospital of Zhengzhou University, Zhengzhou, China; ^2^Henan Key Laboratory for Helicobacter Pylori and Digestive Tract Microecology, The Fifth Affiliated Hospital of Zhengzhou University, Zhengzhou, China; ^3^College of Traditional Chinese Medicine, Fujian University of Traditional Chinese Medicine, Fuzhou, China; ^4^Department of Neurology, The Fifth Affiliated Hospital of Zhengzhou University, Zhengzhou, China; ^5^School of Basic Medical Science, The Shaanxi Key Laboratory of Brain Disorders, Xi’an Medical University, Xi’an, China; ^6^School of Life Sciences, Westlake University, Hangzhou, China; ^7^Henan Provincial Outstanding Overseas Scientists Chronic Liver Injury Workshop, Zhengzhou Key Laboratory of Metabolism-Associated Fatty Liver Disease, The Fifth Affiliated Hospital of Zhengzhou University, Zhengzhou, China

**Keywords:** autism spectrum disorder, dachaihu decoction, 16S rRNA sequencing, metabolomic analysis, gut microbiota

## Abstract

**Background:**

Autism spectrum disorder (ASD) is an early-onset neurodevelopmental disorder, usually accompanied by gut microbiota dysregulation. Gut microbiota homeostasis is considered effective for ASD. Reportedly, *Dachaihu* decoction (DCHD) can efficiently regulate gut microbiota and inflammation. However, the mechanisms underlying the effects of DCHD in the treatment of ASD remain unclear.

**Objective:**

This study investigated the potential effects and mechanisms of DCHD in treating ASD.

**Methods:**

In the animal experiment, propionic acid was administered to construct an ASD rat model. The ASD rats were treated with DCHD, and the efficacy was assessed using the behavioral detections, such as open field test, elevated plus maze test, novel object recognition test. Additionally, the levels of IL-6, TNF-*α*, IL-10, T-SOD, MDA, GSH and CAT were determined using kits, and histological staining was used to evaluate brain morphology. Moreover, tight junction proteins (ZO-1 and occludin) expression levels were evaluated using RT-qPCR, whereas Iba1 expression level was assessed by immunofluorescence staining. The 16S rRNA sequencing and metabolomic analysis of feces revealed the potential targets of DCHD against ASD. In a small human trail, the clinical scales ADOS-2 and Autism Behavior Checklist (ABC) assessed autism severity. Gastrointestinal problems and brain function were evaluated based on food intolerance and event-related potential, respectively.

**Results:**

DCHD significantly improved autism-like behaviors and increased antioxidant enzyme activity, decreased inflammation and enhanced the intestinal barrier by the animal experiment. Furthermore, the DCHD treatment altered the gut microbiota profile, with increased probiotics *Adlercreutzia*, *Parvibacter*, *Turicibacter*, and *Christensenellaceae.* Further, DCHD increased the beneficial metabolite indole-3-acetate and decreased the cognitive impairment-related metabolites asymmetric dimethylarginine and homogentisic acid. Meanwhile, the small clinical trial revealed that DCHD significantly alleviated the core symptoms of ASD, with decreased ADOS-2 and ABC scale scores. DCHD also decreased the levels of specific egg white/yolk and milk IgG antibodies and shortened the MMN and P3b latencies.

**Conclusion:**

This study demonstrated that DCHD may alleviate ASD via inhibiting oxidative stress, reducing inflammation, and modulating the gut microbiota in rats. Combined with human trial, DCHD may be a promising drug for treating ASD. This study provides a scientific rationale for treating mental disorders related to gut microbiota dysbiosis.

## Graphical abstract

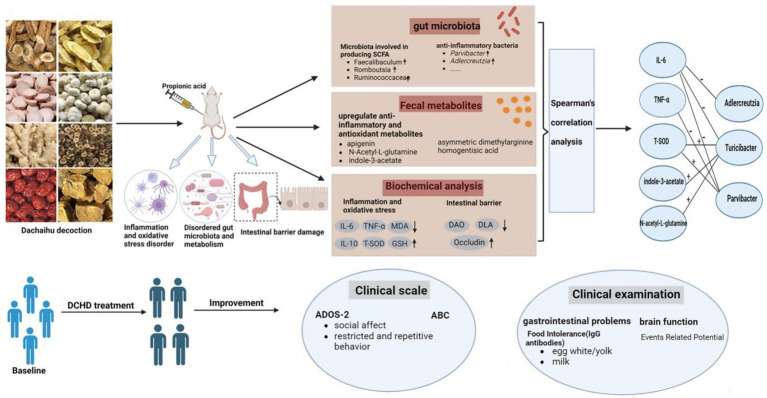

## 1 Introduction

Autism spectrum disorder (ASD) is a group of neurodevelopmental conditions commencing early in life and is characterized by impaired social communication and interactions and stereotyped, repetitive behavior. In western countries, the prevalence of ASD among children and adolescents is 2.76% (1/36) (Maenner et al., 2023). In China, ASD affects 0.7% of children, making it the most prevalent mental disorder (Jiang et al., 2024). The increasing incidence of ASD is a public health concern globally (Bougeard et al., 2024), yet there are no effective drugs to treat ASD. Risperidone and aripiprazole have been approved by the US Food and Drug Administration to treat ASD-associated symptoms in children; however, these drugs may lead to gastrointestinal disorders, irritability, excessive salivation, and other undesirable effects (Biswas et al., 2023). Behavioral psychotherapy is often used to treat ASD, but it is costly and has gradual effects (Qin et al., 2024). Thus, investigating strategies for safely and effectively treating ASD is crucial.

Reportedly, the gut microbiota is an important modulator of host physiology (Fischbach and Segre, 2016), including the endophenotypes associated with ASD (Sherwin et al., 2019), and is known to influence brain physiology and social behavior by participating in the microbiota–gut–brain axis (Sherwin et al., 2019), including immune activation, production of microbial metabolites and peptides, and production of various neurotransmitters and neuromodulators (Sherwin et al., 2019). Many strategies for treating ASD involving interventions in the gut microbiota are being identified, such as fecal microbiota transplantation (FMT), probiotics and prebiotics (Poupard et al., 2024). Human trials (Guidetti et al., 2022; Lin et al., 2024; Mazzone et al., 2024; Wan et al., 2024) have indicated that FMT and probiotics can remarkably improve gastrointestinal symptoms in children with ASD by increasing the abundance of beneficial bacteria. For example, *Limosilactobacillus reuteri* (Mazzone et al., 2024) and *Lactiplantibacillus plantarum* PS128 (Ma et al., 2024) can enhance social behavior in children with ASD, including increasing adaptive social functioning and reducing their social deficits. Reportedly, FMT can reshape the composition and structure of the gut microbiota in patients with ASD, thereby improving gastrointestinal symptoms and ASD-related behavioral symptoms (Kang et al., 2017; Li et al., 2021). Although these methods effectively treat ASD, they are not well promoted because of limitations such as FMT being expensive and complex to perform and probiotics showing individual variability in responses and interactions with medications (Poupard et al., 2024; Tan et al., 2021; Zhang et al., 2023). Thus, exploring novel pharmaceuticals that can preserve the equilibrium of the gut microbiota as a therapeutic approach for ASD is crucial.

Traditional Chinese medicine, recognized for its multitarget approach and low cellular toxicity, comprises numerous promising therapeutics (Wang et al., 2024). Dachaihu decoction (DCHD) comes from the Treatise on Cold Damage and Miscellaneous Diseases (*Shang Han Za Bing Lun*) by *Zhongjing Zhang.* DCHD, a classical formula in ancient China (Cui et al., 2020), effectively regulates mood disorders and depressive symptoms and is effective for exerting anti-inflammatory effects and improving the function of the gastrointestinal tract (Shi et al., 2024). Clinically, DCHD has demonstrated efficacy in treating the “shao-yang signs,” which include symptoms like fever and chills, as well as “yang-ming signs,” characterized by abdominal pain and fullness. In addition, DCHD is also utilized in the treatment of digestive disorders, including inflammatory bowel disease, hepatitis, and gallbladder-related conditions (Duan et al., 2024). Reportedly, DCHD can ameliorate septic gut injury, kidney injury in diabetic mice and high-fat-diet-induced nonalcoholic fatty liver disease by modulating the gut microbiota, glutathione and serum metabolism (Cui et al., 2020; Huang et al., 2023). Hence, DCHD might have great therapeutic potential for ASD by regulating the gut microbiota. However, to our knowledge, no studies have explored the biological basis of DCHD’s action and potential interaction between DCHD and gut microbiota in ASD.

Herein, we investigated the protective effects of DCHD against ASD and the role of crosstalk between DCHD and gut microbiota in preventing ASD using a rat model with propionic acid (PPA)-induced autism. DCHD reversed ASD-associated gut dysbiosis by enriching probiotics and beneficial metabolites, further improving gut–brain barrier function and modulating inflammatory factors. Finally, we elucidated potential interactions between gut microbiota and DCHD metabolites that modulate the efficacy of DCHD in ASD. Altogether, DCHD potentially prevents ASD, the effects of which involve its interaction with gut microbiota.

## 2 Materials and methods

### 2.1 Participants

Children with ASD treated at the Fifth Affiliated Hospital of Zhengzhou University between August 2023 and August 2024 were included. All participants provided written informed consent before enrollment. The clinical trial was approved by the Scientific Ethics and Safety Committee of the Fifth Affiliated Hospital of Zhengzhou University (KY2023077). Patients with ASD conforming to the ASD diagnostic criteria in the Diagnostic and Statistical Manual of Mental Disorders, 5th Edition (DSM-5); whose diagnosis had been made by a senior child psychiatrist based on the patient’s medical history, clinical assessment, and established diagnostic criteria; with 2–6 years of age; and whose parents/guardians had permitted their participation were included. However, those with comorbid other mental illness or cognitive impairment; Abnormal mental behavior caused by organic diseases; Patients with other undiagnosed diseases were excluded. A total of 30 young children (between 24 and 72 months) with ASD were included. The average age was 42.36 (± 14.71) months.

### 2.2 Clinical assessment

The characteristics and severity of the clinical symptoms in children with ASD were evaluated using Autism Diagnostic Observation Schedule (ADOS) and Autism Behavior Checklist (ABC). The ADOS scale was divided into two modules: social affect (SA) and restricted and repetitive behavior (RRB). Each module had a corresponding cutoff score.

### 2.3 Food intolerance

Upon enrollment of the patients, 2 mL of fasting venous blood was collected in the morning using an EDTA-K_2_ anticoagulant tube and centrifuged at 3,500 rpm for a duration of 15 min. The obtained supernatant was stored in a freezer at −80°C. The concentrations of 14 food-specific IgG antibodies were determined using ELISA at the clinical laboratory of the Fifth Affiliated Hospital of Zhengzhou University. The obtained results were categorized into positive groups (+, >50 U/mL) and negative groups (−, <50 U/mL).

### 2.4 Event-related potential

Patients were admitted within 10 days to undergo an event-related potential test using the Nihon Kohden electromyogram and evoked potential device (MEB-2306C). The test was conducted in accordance with the 10–20 system for electrode placement, which facilitated recording of cerebral electrical activity of patients. Reference electrodes—A1 and A2—were positioned over the left and right earlobes. A ground electrode was situated at the forehead midline. The electrode–skin impedance was maintained below 5 kΩ, and the filtering was 0.5–100 Hz. To assess mismatch negativity (MMN), the latencies of the Cz and Fz waveforms were meticulously documented. The mean of these measurements was calculated, and the related potential evoked by the standard stimulus was subtracted from that evoked by the deviant stimulus. MMN was identified as the most prominent negative phase wave within 100–250 ms. Similarly, for the P300 measurement, the latencies of the Cz and Fz waveforms were recorded. P300 was confirmed as the most significant positive phase wave observed within 250–450 ms poststimulus.

### 2.5 *Dachaihu* decoction and ultrahigh-performance liquid chromatography–mass spectrometry analysis

DCHD were provided by Henan Zhangzhongjing Pharmacy Co., Ltd. for the animal experiment. Its components and their proportions are shown in Table 1. The eight herbs components of DCHD were meticulously measured and combined, subsequently, 500 mL of water was introduced to the blend. The solution was heated vigorously to reach boiling point, after which the heat was reduced to let it simmer gently for a duration of 25 min. The solution was filtered to obtain a 250 mL decoction, and decocted at low heat for 25 min to obtain a 125 mL decoction, which represented a single dose of DCHD (equivalent to 0.79 g/mL herbal materials). The decoction was concentrated at 62.5 mL to obtain two doses of DCHD (equivalent to 1.58 g/mL herbal materials). DCHD was analyzed using ACQUITY UPLC I-Class HF with ACQUITY UPLC HSS T3 (100 mm × 2.1 mm, 1.8 μm). Mobile phase A was 0.1% formic acid–water, whereas mobile phase B was acetonitrile, meticulously conducted in accordance with the predefined elution parameters. The main mass spectroscopy conditions were as follows: spray voltage 3,000 V (−) and 3,800 V (+), mass range 125–1,000 m/z, Aux. gas heater temperature 350°C; sheath gas flow rate 35 Arb; and Aux. gas flow rate 8 Arb. The PDA scanning range was 210–400 nm. The process was conducted by Lu-Ming Biotech Co., Ltd. (Shanghai, China).

**Table 1:** The information sheet of *Dachaihu* decoction.

Generic name	Botanical name	Medication batch number	Weight (g)
Chaihu	*Bupleurum chinense* DC.	211,203	15
Huangqin	*Scutellaria baicalensis* Georgi	231,001	9
Zhishi	*Citrus aurantium* L.	22,110,301	9
Shaoyao	*Paeonia lactiflora* Pall.	230,901	9
Ban Xia	*Pinellia pedatisecta* Schott	230,701	9
Dahuang	*Rheum officinale* Baill.	220,901	6
Shengjiang	*Zingiber officinale* Roscoe	/	15
Dazao	*Ziziphus jujuba* Mill	230,902–1	12

### 2.6 Animal experiment

A total of 30 healthy male SD rats (bodyweight 150 ± 50 g) were purchased from the Experimental Animal Center of Zhengzhou University. They were housed in the animal room of the medical campus of Zhengzhou University of Chinese Medicine (temperature 20°C–22°C, humidity 50–60%), with a 12-h light/dark cycle, and all rats had unrestricted access to food and water. This experiment was approved by the Medical and Experimental Animal Ethics Committee of Academy of Medical Sciences of Zhengzhou University [ZZU-LAC20221111(04)]. The rats were randomly assigned to five groups: control, ASD model, DCHD only, DCHD at a low dose (DCH_L), and DCHD at a high dose (DHD_H) (*n* = 6 per group). All groups, except for control and DCHD-only, were subcutaneously administered with PPA (250 mg/kg/day, pH 7.4) for 5 days to induce ASD (Benitah et al., 2023). The daily doses of the DCH_L and DCH_H groups were 4 and 16 g/kg body weight, respectively (Cui et al., 2020). Meanwhile, the control and DCHD-only groups were given normal saline. After 4 weeks of the DCHD treatment, the rats underwent behavioral analysis for 4 weeks. Subsequently, the feces were collected and the rats were sacrificed.

### 2.7 Behavioral assessments

A researcher, blinded to the group assignments, conducted the behavioral evaluations. ASD-like behavior was scored using the Smart 3.0 tracking software (PanLab, United States). The behavioral assessments were carried out during the day between 09:00 and 18:00 h.

### 2.8 Open field test

For this test, the rats were placed in a 100 cm × 100 cm × 40 cm black plastic box. The box was divided into central and peripheral areas. An overhead camera system was used to record its path, and the Smart3.0 software was employed to determine the frequency of entries and the time duration within the central area. The open field test (OFT) is common assay for evaluating both anxiety-like and motor behaviors for animals (Kraeuter et al., 2019).

### 2.9 Three-chamber social test

The experiment was carried out in a connected transparent three-chamber chamber, a classic way of assessing social skills in rats (Buffington et al., 2016). Prior to the test, the rat is placed in the test environment for acclimatization for 2 h. For the social experiment, the left metal box was placed into the stranger rat 1, and the experimental rat was observed for 10 min. The Smart3.0 software analysis system was used to calculate the residence time of the tested rat in S1 and the empty cage (E1). In the social preference experiment, the left metal box was placed into the stranger rat 2 (S2) and the S1. The test time was 10 min, and the residence time of the tested rats on both sides was analyzed.

### 2.10 Elevated plus maze test

According to the experiment, each rat was positioned on the central platform (15 cm × 15 cm), facing the direction of the open arm (45 cm × 15 cm). The activities of each rat in both the open arm and the closed arm (45 cm × 15 cm × 30 cm) were meticulously observed. Conducted in dim lighting and a quiet environment, the experiment lasted for 5 min. During this period, the movement track of each rat in the elevated cross maze, as well as the time spent and distance traveled in the open arm, were carefully monitored. The elevated plus maze (EPM) experiment is designed to investigate the anxiety state of animals by utilizing their innate tendency to explore new environments and their conflicting behavior stemming from fear of the high, open, and suspended spaces.

### 2.11 Novel object recognition test

For this experiment, the rats were initially allowed to acclimate to the surroundings by being placed in the laboratory for 1 day before the test. The novel object recognition test consisted of three stages. First, two identical objects [old object (O) and familiar object (F)] were placed on opposite sides of the testing box of 60 × 60 × 60 cm^3^, each approximately 10 cm from the walls of box. The rats were placed the box and allow them to explore freely for a 5 min period before removing them. Following each rat was tested, clean the box with 75% ethanol to eliminate any residual odor. Twenty-four hours later, one of the familiar objects in the test box was randomly substituted with a novel object (N) of comparable size but differing in shape and color, while the positions of both objects remained unchanged. The rats were then placed back into the experimental box to explore for 5 min before being removed.

### 2.12 Biochemical analysis

Serum samples were collected following centrifugation at 3,500 rpm for 15 min. The GSH, T-SOD, MDA, AST, ALT, and BUN levels were measured using the corresponding biochemical kits (Nanjing Jiancheng Institute of Biotechnology, Nanjing, China). The level of IL-6, IL-10, and TNF-*α* also were measured by the rat ELISA kits (Fankel, Shanghai, China), per the manufacturer’s instructions. Moreover, a microplate reader was used to measure absorbance (PerkinElmer, Germany).

### 2.13 Hematoxylin and eosin staining and immunohistochemistry

For histopathological analysis, samples from the liver, kidney, and hippocampus were routinely fixed in 4% paraformaldehyde and then embedded in paraffin wax, cut into 5-μm-thick slices, and subsequently subjected to dehydration, embedding, and sectioning. Further, the sections were observed using a Nikon imaging system (TS2). For immunohistochemistry, paraffin-embedded sections of brain tissue were heated at 60°C, dewaxed with water, subjected to antigen retrieval, and closed at room temperature. The primary anti-Iba1 (17,198, 12,000; Cell Signaling Technology) was added, followed by incubation at 4°C overnight. Subsequently, rinse them with PBS five times, allowing 5 min for each rinse. Afterwards, rabbit secondary antibody (HRP-labeled) (Servicebio, China) was added, followed by incubation in the dark for 1 h, rinsing with PBS, color development by DAB, dehydration, and sealing. The immunohistochemistry samples were photographed under an upright microscope and analyzed using ImageJ (version: 1.8.0.112).

### 2.14 Immunofluorescence analysis

The dewaxed coronal sections were subjected to antigen retrieval. The primary antibody rabbit antirat Iba1 (Cell Signaling Technology, 17,198), prepared at a 1:100 ratio with PBS, was added dropwise to the sections, which were then incubated at 4°C overnight. The secondary antibody anti-rabbit IgG was diluted in 4% BSA in PBS and incubated at room temperature for 1 h. The cell nucleus was stained with DAPI at room temperature for 10 min, avoiding light. Fluorescence images were obtained using a confocal laser scanning microscope (DMI8 DFC7000T; Leica Microsystems).

### 2.15 mRNA expression analysis by RT-qPCR

Total RNA was extracted from the colon using Trizol reagent (Thermo Fisher) and processed per the PrimeScript RT Reagent Kit (Vazamy, Nanjing, China) instructions to synthesize cDNA. qPCR was performed in an RT-qPCR instrument (MA-Smart) per the SYBR Premix Ex Taq II Reagent (Vazamy, Nanjing, China) instructions. GAPDH was used as an internal reference gene, and the 2^–ΔΔCT^ relative quantitation method was used to analyze gene expression. The used primers were synthesized by Shanghai Shenggong Technology Co., Ltd., and Table 2 contains the primers listed for qRT-PCR analysis.

**Table 2:** Sequences of primers used in the study.

Gene	Forward primer (5’- > 3’)	Reverse primer (5’- > 3’)
GAPDH	CCTGGAGAAACCTGCCAAG	CACAGGAGACAACCTGGTCC
Occludin	AGCGAAGAGTACATGGCTGC	TCACTTCTCCAGCAACCAGC
ZO-1	GGAGCGGGGACAAGATGAAG	GAGGATGGAGTTACCCACAGC

### 2.16 16S rRNA sequencing and bioinformatic analysis

Approximately 1.0 g of midstream fecal samples were collected from PPA-treated rats and preserved at a temperature of −80°C for subsequent analysis. DNA was extracted from these samples using the Tiangen Biochemical Technology Co., Ltd. (Beijing, China) Fecal Genome DNA Extraction Kit (DP328-02). The V3-V4 regions of the 16S rRNA gene were targeted for amplification using the primers 341F (CCTACGGGNGGCWGCAG) and 785R (GACTACHVGGGTATCTAATCC). Following amplification, the PCR products were purified and subjected to agarose gel electrophoresis to isolate the amplicon. The target DNA fragment was then excised, purified, and prepared for sequencing. The purified DNA libraries were sequenced on the Illumina Novaseq 6,000 platform using a paired-end (PE250) sequencing strategy. Post-sequencing, the cutadapt software was employed to remove primer sequences from the raw reads, and vsearch was used to merge the paired-end sequences. Sequences with a similarity of 97% or greater were clustered into operational taxonomic units (OTUs). These OTUs, representing the core sequences within each cluster, were then utilized for taxonomic annotation and further classification analysis. Alpha diversity was determined using QIIME2. Beta diversity was analyzed by principal coordinate analysis (PCoA). Linear discriminant analysis (LDA) coupled with effect size (LEfSe) was applied to evaluate the taxa with differences in abundance between the groups. Bacterial groups exhibiting an LDA score of 2.00 or higher were deemed to be significantly abundant within a specific group. The 16S rRNA analysis data presented in the study are deposited in the National Center for Biotechnology Information Sequence Read Archive database, accession numbber PRJNA1131930; The metabolomic analysis data presented in the study are deposited in the MetaboLights database (accession code: MTBLS3435).

### 2.17 Metabolomic analysis

The 50 mg fecal sample was precisely weighed and transferred into a 2 mL centrifuge tube. To this, 600 μL of methanol containing 2-chloro-L-phenylalanine was added, and the mixture was vortexed for 30 s to ensure thorough mixing. Subsequently, 100 mg of glass beads were introduced into a tissue grinder, and the sample was ground at a frequency of 60 Hz for 90 s. Following grinding, the sample was subjected to ultrasonication at room temperature for 10 min to further aid in the extraction process. After ultrasonication, the sample was centrifuged at 12,000 rpm for 10 min at a temperature of 4°C to separate the supernatant from the solid particles. The supernatant was then passed through a 0.22 μm membrane filter to remove any remaining particulates and collect the resulting filtrate. The LC/MS system for untargeted metabolomic analysis comprised Waters Acquity I-Class PLUS ultrahigh-performance liquid tandem Waters Xevo G2-XS QT high-resolution mass spectrometer. Raw data collected using MassLynx were processed by R XCMS (v3.12.0) software. Metabolites with RSD > 30% in QC samples were filtered and used for subsequent data analysis. Subsequently, the identified compounds were used as queries for searches for classification and pathway information in HMDB (Wishart et al., 2007), MassBank (Horai et al., 2010), KEGG (Ogata et al., 1999), LipidMaps (Sud et al., 2007), mzcloud (Abdelrazig et al., 2020), and the metabolite database built by Panomix Biomedical Tech Co., Ltd. (Shuzhou, China). PCoA was used to judge the repeatability of the samples within groups. The R package ropls was used to perform OPLS-DA modeling, and 200× permutation tests were performed to confirm model reliability. The significantly enriched KEGG pathways among the differential metabolites were calculated using the hypergeometric distribution test. The 16S rRNA analysis data presented in the study are deposited in the National Center for Biotechnology Information Sequence Read Archive database, accession numbber PRJNA1131930; The metabolomic analysis data presented in the study are deposited in the MetaboLights database (accession code: MTBLS3435).

### 2.18 Statistical analysis

All data are presented as mean ± SD. Two groups were compared using unpaired Student’s t-test or Mann–Whitney U test. Categorical variables were compared between two groups using the χ^2^ or Fisher’s exact test. Multiple groups were compared by Wilcoxon’s test or one-way ANOVA. All statistical analyses were conducted using GraphPad Prism (version 9.5.0) or R package. Pearson’s correlation analysis was used to determine the correlation between different parameters. Spearman’s correlation analysis was used to explore the relationships of gut microbiota exhibiting differential expression between groups with social behaviors, serum physiological measures, and differentially expressed metabolites. All *p* values were two-sided, and *p* < 0.05 was regarded as indicative of statistical significance (*p < 0.05, **p < 0.01, ***p < 0.001, ****p < 0.0001, ns, not significant).

## 3 Results

### 3.1 Ultrahigh-performance liquid chromatography–mass spectrometry chromatogram analysis of multiple active ingredients in DCHD

Ultrahigh-performance liquid chromatography–mass spectrometry was employed to analyze the 11 primary active ingredients of DCHD (Cui et al., 2020). From the positive-ion mode results, 1, 2, 3, 4, 5, 6, 7, and 8 were identified as synephrine, albiflorin, paeoniflorin, naringin, neohesperidin, baicalin, saiko saponin A, and [6]-gingerol, respectively (Supplementary Figure S1B). From the negative-ion mode results, 1, 2, and 3 were identified as succinic acid, hesperidin, and emodin, respectively (Supplementary Figure S1C).

### 3.2 *Dachaihu* decoction improved autism-like behavior in rats with propionic acid-induced autism spectrum disorder

**Figure 1:**
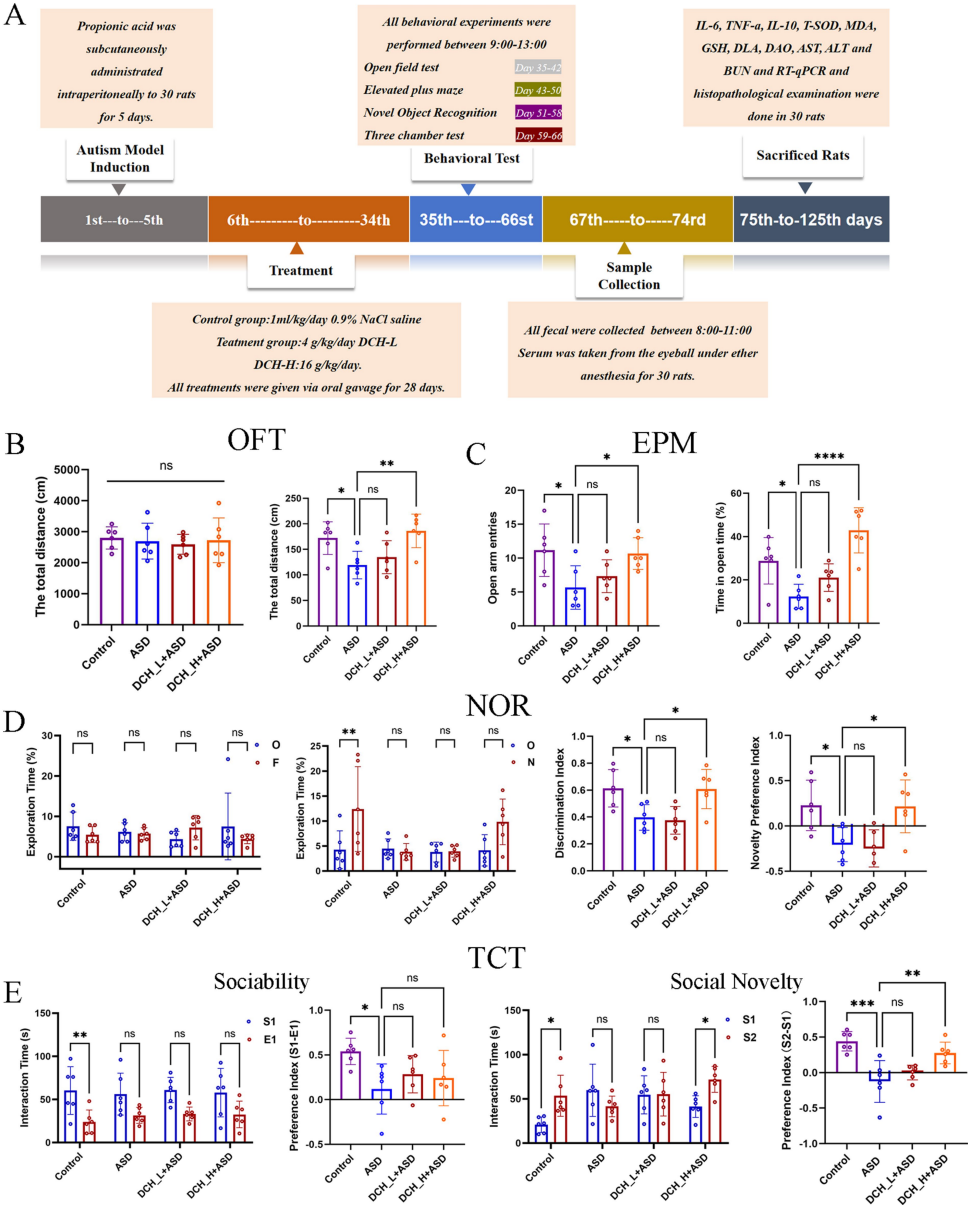
Experimental protocol and results of rat behavioral experiments. **(A)** Schematic of the animal study protocol. **(B)** Results of OFT in rats after 4 weeks of DCHD treatment. **(C)** Results of EPM test in rats after 5 weeks of DCHD treatment. **(D)** Results of NOR test in rats after 6 weeks of DCHD treatment. **(E)** Results of TCT in rats after 7 weeks of DCHD treatment.One-way ANOVA followed by Tukey’s *post hoc* test was used for multiple pairwise comparison. *p < 0.05, **p < 0.01, ***p < 0.001, ns, not significant.

To explore the therapeutic effect of DCHD in ASD, several social behavior tests—open field test (OFT), elevated plus maze (EPM), novel object recognition (NOR) test, and three-chamber sociability tests(TCT)—were conducted (Figure 1A). Compared with the control, PPA-treated rats displayed impaired ASD-related behaviors, as reflected by the decreased total distance of movement (Figure 1B), fewer open-arm entries and less time spent in open arms (Figure 1C), the poor recognition of novelty (Figure 1D), and lack of preference for the stranger and novel rats in the sociability and social novelty tests (Figure 1E); this was consistent with previous results(Liu et al., 2024). However, after 4 weeks of DCH_H treatment, the total traveled distance had significantly increased (*p* < 0.05) in the OFT (Figure 1B). Meanwhile, in the EPM test, the rats treated with DCH_H exhibited more numerous entries and more time to the open arms than those the ASD group (Figure 1C), suggesting decreased anxiety. Moreover, in the NOR test (Figure 1D), rats in the DCH_H group were more inclined to explore new objects than those in the ASD group (*p* < 0.05), indicating improved learning and memory skills. In the three-chamber sociability test (Figure 1E), rats with autism treated with DCH_H possessed better socialization skills, with increased exposure to new rats and exhibiting strong socialization abilities (*p* < 0.05), suggesting that DCH_H administration improved socialization in autistic rats. For all behavioral tests, no significant differences were found between the DCH_L treatment and ASD group. Thus, 4 weeks of treatment with DCH_H but not DCH_L reversed the social deficits in rats with PPA-induced autism.

### 3.3 *Dachaihu* decoction attenuated/mitigated inflammation, oxidative stress, and intestinal permeability of autistic rats

**Figure 2:**
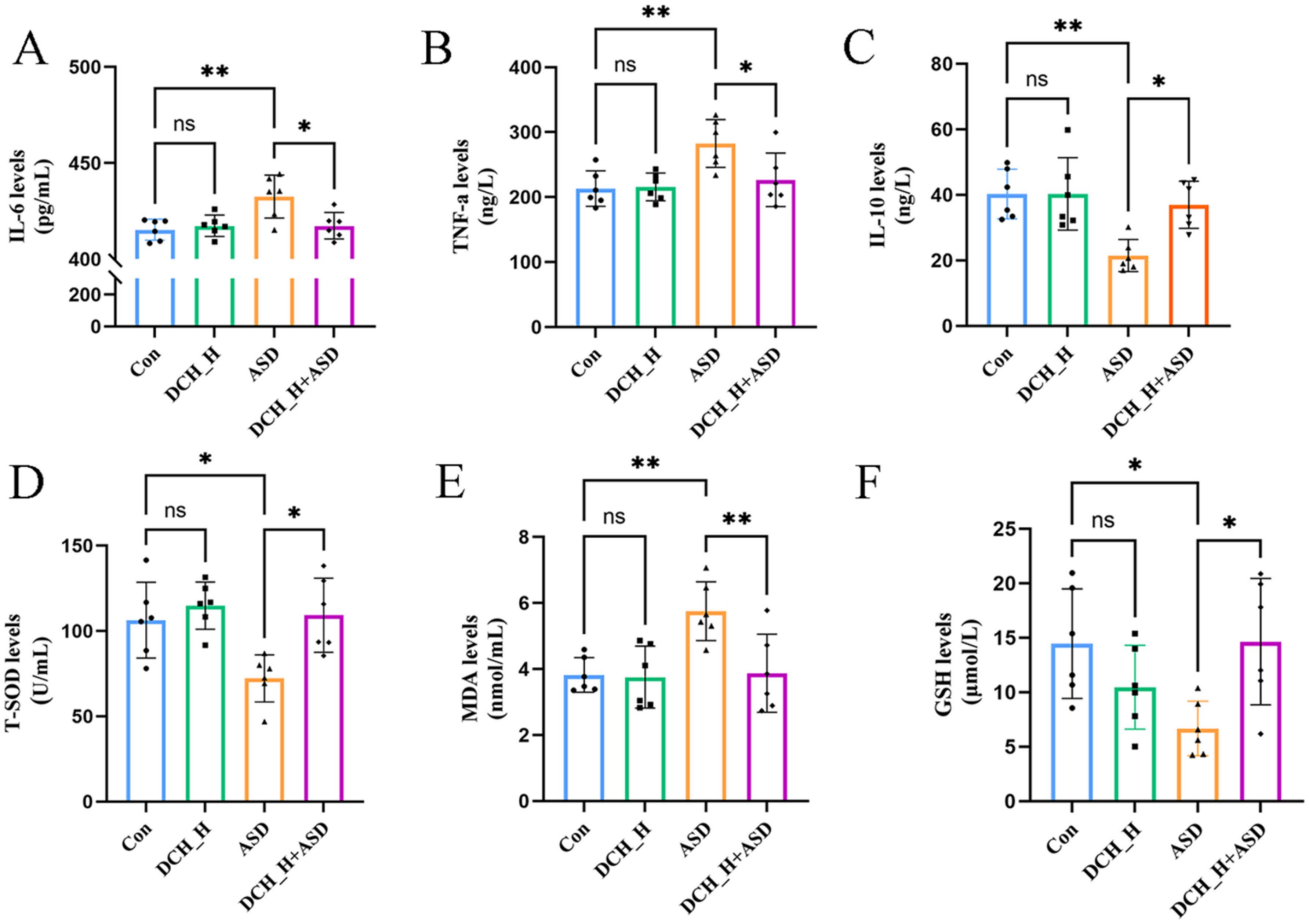
*Dachaihu* decoction effectively relieved inflammation, oxidative stress, and intestinal permeability in rats with PPA-induced autism. **(A–F)** Serum levels of IL-6, TNF-*α*, IL-10, T-SOD, MDA, and GSH.One-way ANOVA followed by Tukey’s post hoc test was used for multiple pairwise comparison. *p < 0.05, **p < 0.01, ***p < 0.001, ns, not significant.

The key indicators of oxidative stress (GSH, T-SOD, and MDA) and levels of inflammatory factors (TNF-*α*, IL-6, and IL-10) were determined to evaluate the pharmacodynamic effect of DCHD for improving ASD. There were significantly increased in IL-6, TNF-α, and MDA levels (*p* = 0.0044, 0.0073, and 0.0080, respectively) in the PPA-induced ASD group (432.60 ± 11.18 pg/mL, 282.66 ± 36.96 ng/L, and 5.75 ± 0.89 nmol/mL) compared with those in the control group (415.34 ± 5.48 pg/mL, 213.20 ± 27.24 ng/L, and 3.82 ± 0.53 nmol/mL). Meanwhile, the induction of autism by PPA resulted in significantly decreased in the serum levels of IL-10 (*p* = 0.0031), GSH (*p* = 0.0322), and T-SOD activity (*p* = 0.0207) (21.48 ± 4.90 ng/L, 6.68 ± 2.51 μmol/L, and 72.27 ± 13.85 U/mL) compared with those in the control group (40.25 ± 7.57 ng/L, 14.45 ± 5.03 μmol/L, and 106.38 ± 22.22 U/mL) (Figures 2A–F). With the antioxidant and anti-inflammatory effects of DCHD, IL-6 and TNF-α levels were significantly decreased after 4 weeks of DCH_H treatments (*p* = 0.0119), compared with the findings in the ASD group. Furthermore, DCH_H treatment significantly reversed the changes in the serum levels of IL-10, MDA, and GSH (*p* = 0.0137, 0.0349 and 0.0295), as well as T-SOD activity (*p* = 0.0113), relative to those in the ASD group. Moreover, theeffects of DCHD on the liver and kidney were explored. Biomarkers (serum AST, ALT, and BUN) indicated that DCHD was not hepatotoxic or nephrotoxic to the rats (Supplementary Figures S2B–D). Furthermore, there were no obvious histopathological distinctions between the DCHD treatment and control group in liver and kidney sections (Supplementary Figure S2A).

**Figure 3:**
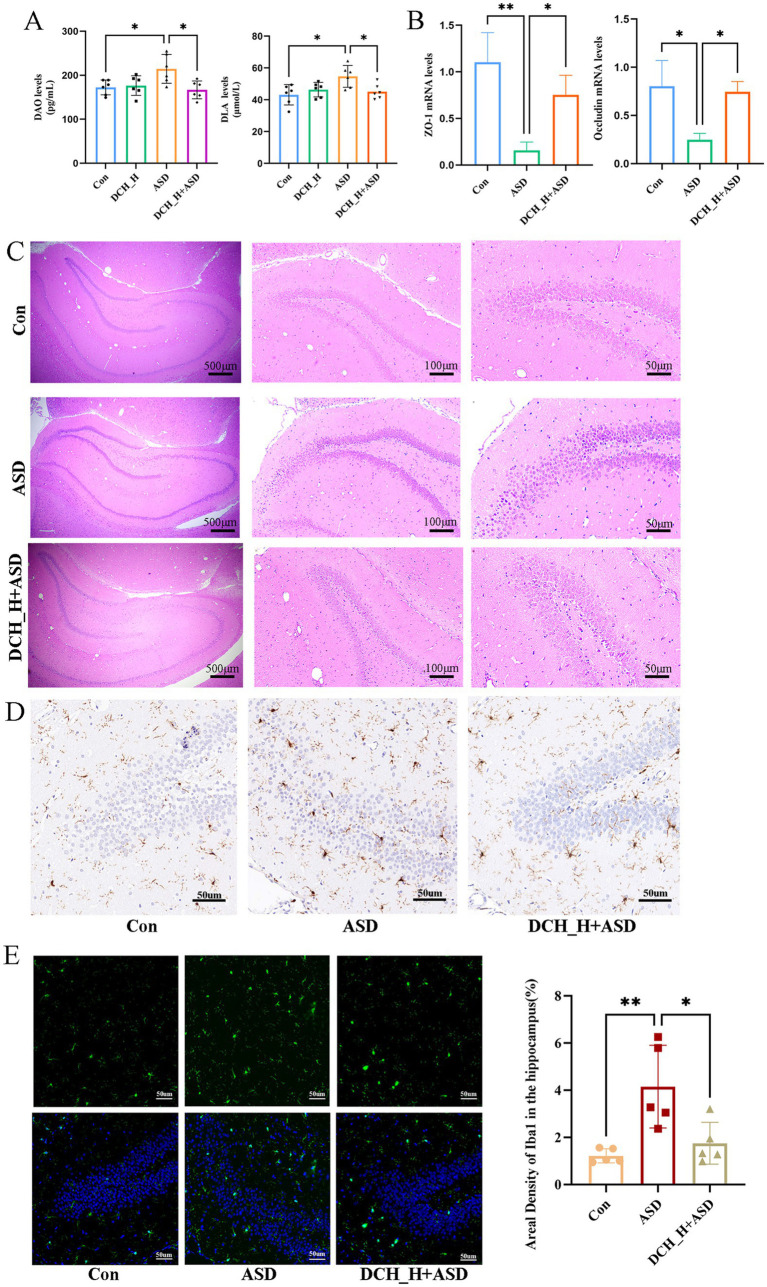
*Dachaihu* decoction effectively relieved intestinal permeability in rats with PPA-induced autism. **(A)** DAO and DLA levels. **(B)** ZO-1 and occludin mRNA expression in colon (*n* = 3, each group) assessed by RT-qPCR. **(C-E)** Brain tissue sections underwent hematoxylin and eEosin (H&E) staining, and immunohistochemical and immunofluorescence studies for histopathological analysis (*n* = 5, each group). One-way ANOVA followed by Tukey’s post hoc test was used for multiple pairwise comparison.*p < 0.05, **p < 0.01, ***p < 0.001, ns, not significant.

Increased gut barrier permeability can translocate various luminal contents into the circulatory system, potentially triggering immune responses and neuroinflammation, which could in turn affect brain function and behavior(Chelakkot et al., 2018). This can also activate the microglia of the central nervous system and release proinflammatory cytokines(Alexandrov et al., 2020; Kelly et al., 2015). To evaluate whether DCHD can impact gut barrier function, we tested serum DAO and DLA, biomarkers of gut barrier integrity, that significantly increased upon the induction of autism by administering PPA (DAO, 214.60 ± 32.71 pg/mL, *p* = 0.0283; DLA, 54.69 ± 6.88 μmol/L, *p* = 0.0110) compared with the control group (DAO, 142.47 ± 16.78 pg/mL; DLA, 43.13 ± 6.41 μmol/L). However, DCH_H treatment markedly decreased the DAO and DLA levels (DAO, *p* = 0.0118; DLA, *p* = 0.0405; Figure 3A). Accordingly, the expression of ZO-1 and occludin, declined in the autistic rats, but was elevated in the DCH_H-treated rats, as determined by RT-qPCR (Figure 3B). These findings indicated that DCHD might contribute to restoring the function of the gut barrier.

Hippocampal lesions are reportedly associated with the social memory deficits in ASD (Banker et al., 2021). Herein, hematoxylin and eosin staining showed that the ASD group exhibited a loose arrangement of neurons and necrotic neurons in the DG region of the hippocampus, in contrast to the control group, whereas DCH_H treatment slowed down cell atrophy, as evidenced by more tightly arranged neurons (Figure 3C). Consequently, the number of microglia in the hippocampal DG regions was significantly increased in rats with PPA-induced autism but was decreased by DCHD treatment (Figures 3D,E). Thus, the number of microglia in the hippocampus, density of Iba-1, and branches of the microglia were consistently restored by DCH_H, as revealed by immunofluorescence staining (*p* < 0.05). These results suggested that DCHD might improve hippocampal function through the gut–brain axis.

### 3.4 *Dachaihu* decoction modulated propionic acid-induced gut dysbiosis in autistic rats

**Figure 4:**
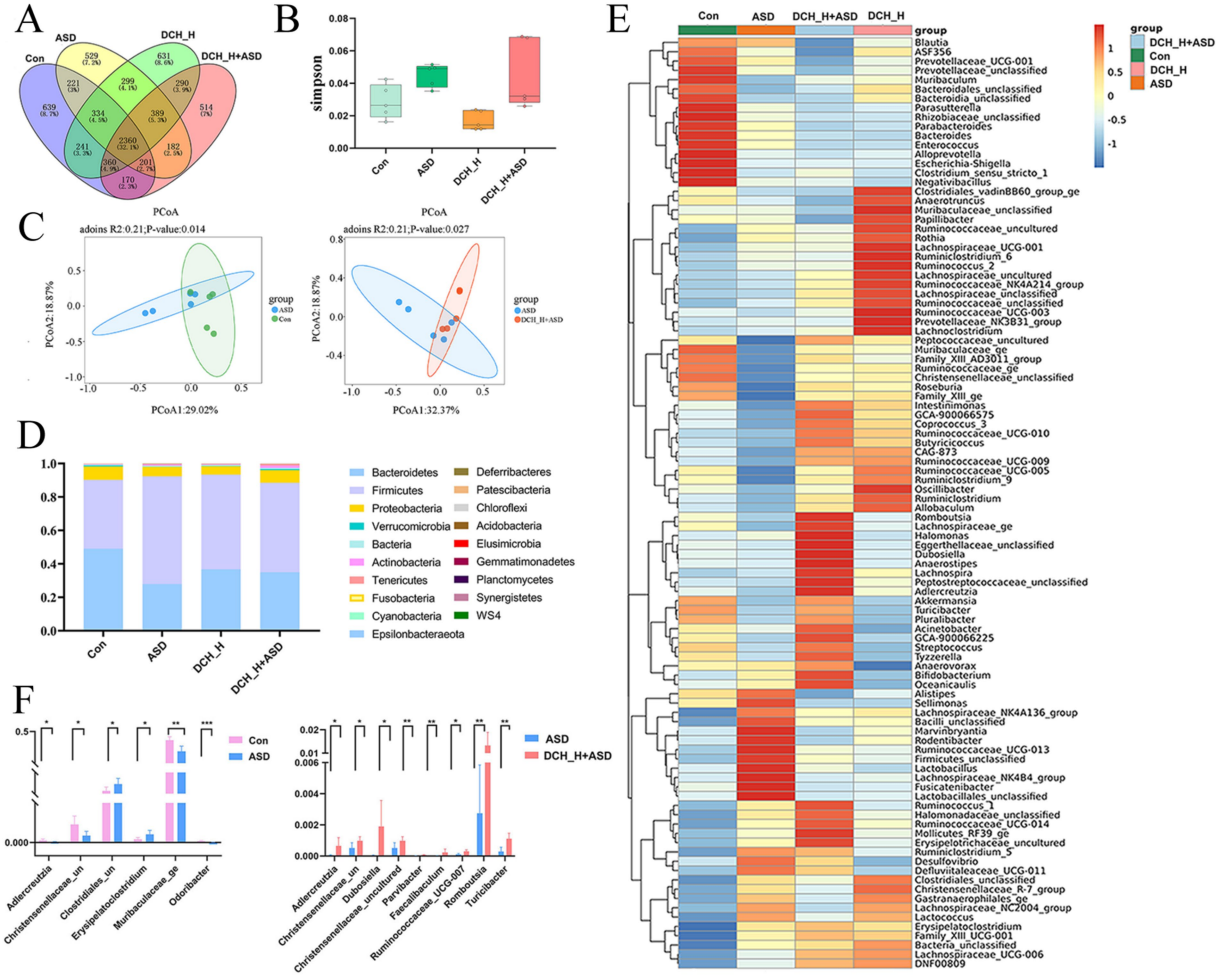
DCHD regulated gut microbiota dysbiosis in rats with PPA-induced autism after 4 weeks of treatment. **(A)** Venn diagram showing the overlap of OTUs among the four groups. **(B)** Simpson index (α-diversity) and **(C)** Principal component analysis (PCoA) (*β*-diversity) of the gut microbiota. **(D)** Histogram of relative abundance of the gut microbiota at the phylum level. **(E)** Heatmap of gut microbial genera among the four groups. **(F)** Bacteria with differential abundance between the ASD and control groups, and between the DCHD-treated and ASD groups. One-way ANOVA followed by Tukey’s post hoc test was used for multiple pairwise comparison. *p < 0.05, **p < 0.01, and ***p < 0.001.

Alterations in the gut microbiota reportedly correlates with behavioral problems in patients with autism(Chen et al., 2022). To determine if DCHD intervention changs the gut microbiota thereby involved in improved ASD-like behaviors, we profiled the gut microbiota composition of rats through 16S rRNA sequencing. A Venn diagram at the OTU level shows 2,360 common OTUs among the four groups, with each group harboring unique OTUs (Figure 4A). Results from the PCoA of *β*-diversity plots indicate that DCH_H, without impacting microbial ɑ-diversity (Figure 4B), modified the structural composition of the gut microbial community (ASD vs. Control, *p* = 0.018; DCH_H vs. ASD, *p* = 0.031 by Adonis analysis; Figure 4C). At the phylum level, the *Firmicutes/Bacteroidetes* ratio was significantly higher in rats with ASD (2.8912 ± 1.6552) than in control rats (0.9075 ± 0.4564), whereas the *Firmicutes/Bacteroidetes* ratio was partially restored (1.9489 ± 1.2763) after 4 weeks of DCH_H treatment (Figure 4D). The LEfSe revealed that the ASD rats were characterized by high levels of *Pasteurellaceze*, *Pasteurellales*, and *Rodentibacter*, while DCH_H treatment was associated with high levels of *Peptostreptococcaceae*, *Romboutsia*, *Tyzzerella*, *Parvibacter*, and *Adlercreutzia* (Supplementary Figure S3). A heatmap of gut microbial genera showed that DCH_H holistically altered the PPA-induced composition of microbiota (Figure 4E). Notably, DCHD treatment reversed the decreases in the beneficial bacteria *Adlercreutzia* and *Christensenellaceae*_uncultured in the ASD group (Figure 4F). Meanwhile, the relative abundance of the beneficial bacteria *Muribaculaceae_ge* and *Odoribacter* significantly decreased in the PPA-induced rats group compared with the control group (Figure 4F). Conversely, genera such as the harmful bacteria *Erysipelatoclostridium* and *Clostridiales*_unclassified increased in the ASD group (Figure 4F). Furthermore, the levels of the probiotics *Faecalibaculum*, *Dubosiella, Parvibacter*, *Romboutsia*, *Turicibacter*, and *Ruminococcaceae*_UCG-007 were significantly increased in the ASD rats after DCHD treatment (Figure 4F). Considerably, DCHD treatment affected the gut microbiota profile and optimized the bacterial composition in ASD rats.

### 3.5 *Dachaihu* decoction corrects gut microbiota metabolite imbalances/modulates gut metabolites in autistic rats

**Figure 5:**
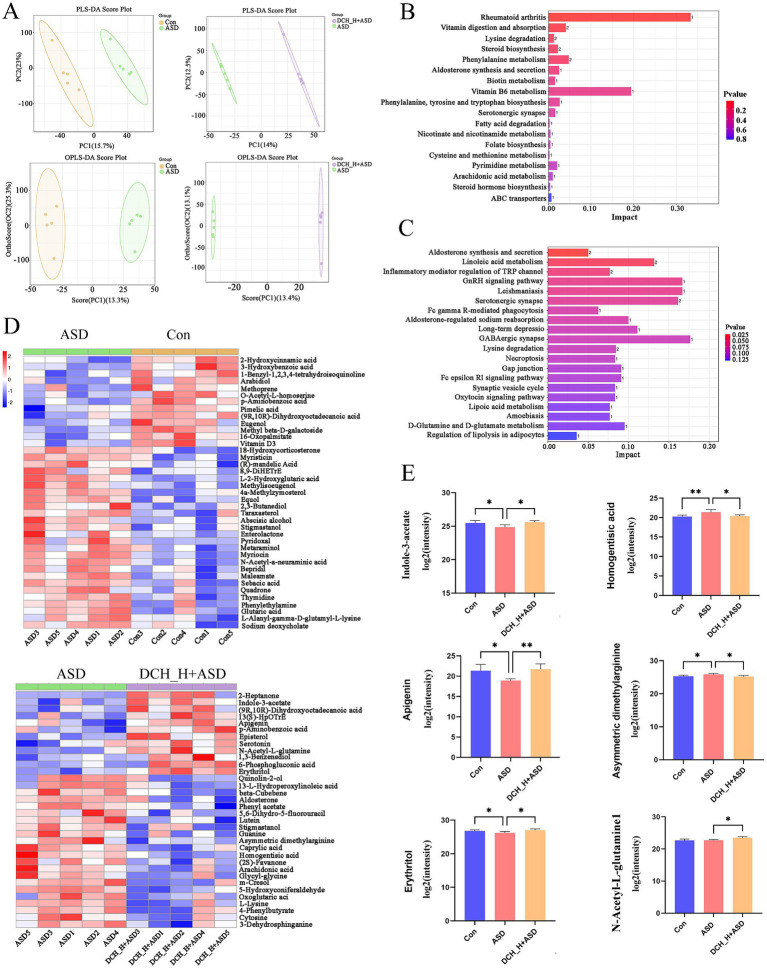
DCHD regulated metabolic processes in gut microbiota of rats with PPA-induced autism. **(A)** Clustering analyses of partial least-squares discriminant analysis (PLS-DA) and orthogonal partial least-squares discriminant analysis (OPLS-DA). **(B)** KEGG enrichment analysis of differential metabolites between the Con and ASD groups. **(C)** KEGG enrichment analysis of differential metabolites between the DCH_H and ASD groups. **(D)** Different metabolites between the ASD and control groups and different metabolites between the ASD + DCH_H and ASD groups. **(E)** Gut metabolism is regulated by DCHD, *p < 0.05, **p < 0.01. Metabolites with >1-fold change, with VIP > 1 and *p* < 0.05 (*t*- test).

Gut microbiota can regulate the development and function of the host nervous system through its metabolites (Xiao et al., 2022; Zheng et al., 2021). Thus, we performed metabolomic analysis of the gut microbiota. The fecal samples from distinct groups were largely separated by partial least-squares discriminant analysis (PLS-DA) and Orthogonal Partial Least Squares Discriminant Analysis (OPLS-DA) (Figure 5A), suggesting distinct metabolic modes. KEGG enrichment analysis of differential metabolites between the Con and ASD groups and between the DCH_H and ASD groups (Figures 5B,C). Subsequent bioinformatic analysis identified 20 significantly enriched KEGG pathways between the DCH_H and ASD groups (Figure 5C), among which aldosterone synthesis and secretion (ID: rno04925, *p* = 0.021), Linoleic acid metabolism (ID: rno00591, *p* = 0.033), Inflammatory mediator regulation of TRP channels (ID: rno04750, *p* = 0.049), Serotonergic synapse (ID: rno04726, *p* = 0.068), and Lysine degradation (ID: rno00310, *p* = 0.092) were notable for containing the most differentially expressed metabolites, and among which GABAergic synapse (ID: rno04727, *p* = 0.088712694) showed the highest significance. Meanwhile, 39 distinct metabolites (FC > 1, VIP > 1, and *p* < 0.05) were identified between the PPA-induced rats group and control group, which included 26 and 13 metabolites up- and down-regulated in ASD (Figure 5D). Meanwhile, 34 distinct metabolites were identified between the DCHD-treated and ASD groups (FC > 1, VIP > 1, and *p* < 0.05), 12 and 22 of which were up- and down-regulated, respectively (Figure 5D). Notably, the findings on the differential metabolites in the feces revealed that DCHD upregulated metabolites such as indole-3-acetate, apigenin, erythritol, and N-acetyl-L-glutamine, while down regulating metabolites such as asymmetric dimethylarginine, homogentisic acid, m-cresol and caprylic acid, and arachidonic acid (Figure 5E).

### 3.6 Correlations of the altered gut microbiota with behaviors, serum biochemical parameters, and gut metabolites in autistic rats

**Figure 6:**
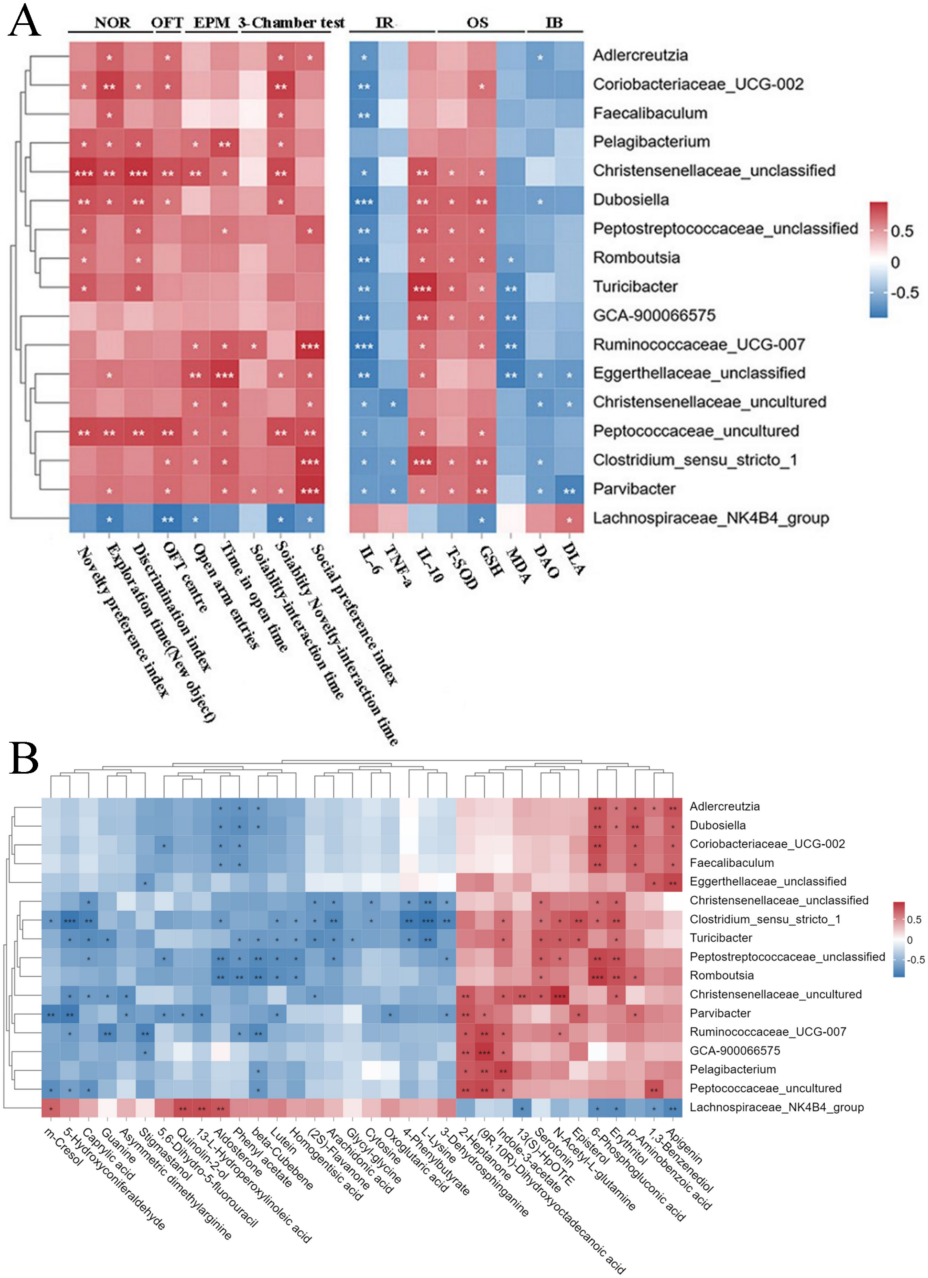
Spearman’s correlation analysis. **(A)** Correlations between the differential genera and ASD-related behaviors and serum biochemical parameters of ASD rats after DCHD treatment.+ **(B)** Correlations between the differential genera and metabolites in feces of ASD rats after DCHD treatment. The different genera detected in 16S rRNA sequencing data between DCHD_H and ASD groups (Metastats analysis, *p* < 0.05). IR, inflammatory response; OS, oxidative stress; IB, intestinal barrier. Metabolites with >1-fold change, with VIP > 1 and *p* < 0.05 (*t*- test) between DCHD_H and ASD groups. The correlation coefficient is indicated by a color gradient from blue (negative correlation) to brick-red (positive correlation). *p* values were corrected for multiple testing using the Benjamini–Hochberg false discovery rate. *p < 0.05, **p < 0.01, and ***p < 0.001.

Gut microbiota is considered closely related to autistic behaviors (Zarimeidani et al., 2024). Herein, Spearman’s correlation analysis showed that the genera *Parvibacter* and *Dubosiella* were a strong correlation to improved social behaviors, followed by *Romboutsia* and *Christensenellaceae*_uncultured. Such behaviors included the increased total distance of movement, the more open-arm entries and longer time spent in open arms, the better novelty performance, and the improved preference for the stranger rat in the sociability test and for the novel rat in the social novelty test (Figure 6A). Overall, DCHD regulation of social behaviors is linked to specific gut microbiota, suggesting that DCHD can regulate the gut microbiota of PPA-induced rats and improve social behaviors.

Herein, correlations were found between the differential gut microbiota and serum chemical parameters, including inflammatory response indices IL-6, TNF-ɑ, and IL-10; oxidative stress indices T-SOD, GSH, and MDA; and intestinal barrier markers DAO and DLA (Figure 6A). In addition, the results showed that most DCHD-promoted beneficial bacteria, such as *Adlercreutzia*, *Faecalibaculum*, *Dubosiella*, *Romboutsia*, *Christensenellaceae*_unclassified, *Ruminococcaceae*_UCG-007, and *Parvibacter*, were negatively associated with serum IL-6 level. Meanwhile, the levels of *Christensenellaceae*_unclassified, *Dubosiella*, *Peptostreptococcaceae*_unclassified, *Romboutsia*, *Ruminococcaceae*_UCG-007, and *Parvibacter* were positively associated with IL-10, T-SOD, and GSH. Additionally, the genera *Adlercreutzia*, *Dubosiella*, and *Parvibacter* were negatively associated with either serum DAO or DLA content.

The gut microbiota significantly contributes to the regulation of metabolism. To investigate the potential links between the changes in the gut microbiota and metabolites, we used Spearman’s correlation analysis to creat a correlation matrix (Figure 6B). Some bacteria are strongly correlated with many metabolites (cor > 0.5 or cor < −0.5). For example, *Adlercreutzia*, *Coriobacteriaceae*_UCG-002, *Dubosiella*, *Eggerthellaceae_*unclassified and *Faecalibaculum* were positively correlated with apigenin. Meanwhile, *Adlercreutzia, Dubosiella, Romboutsia*, *Turicibacter*, and *Christensenellaceae*_uncultured were positively correlated with erythritol. In addition, *Parvibacter* and *Christensenellaceae* were negatively correlated with asymmetric dimethylarginine. However, *Turicibacter* and *Ruminococcaceae*_UCG-007 were positively correlated with indole-3-acetate. *Parvibacter* was negatively correlated with m-cresol. Furthermore, *Adlercreutzia*, *Faecalibaculum*, and *Dubosiella* were negatively correlated with aldosterone. *Christensenellaceae, Turicibacter, Romboutsia*, and *Peptostreptococcaceae*_unclassified were positively correlated with serotonin. Finally, *Ruminococcaceae*_UCG-007 and *Turicibacter* were positively correlated with N-acetyl-L-glutamine. Therefore, DCHD may improve PPA-induced disordered social behaviors by regulating gut microbiota, potentially by influencing inflammatory response, oxidative stress, intestinal barrier function, and gut microbial metabolism.

### 3.7 *Dachaihu* decoction significantly improves the clinical symptoms of autism spectrum disorder

**Figure 7:**
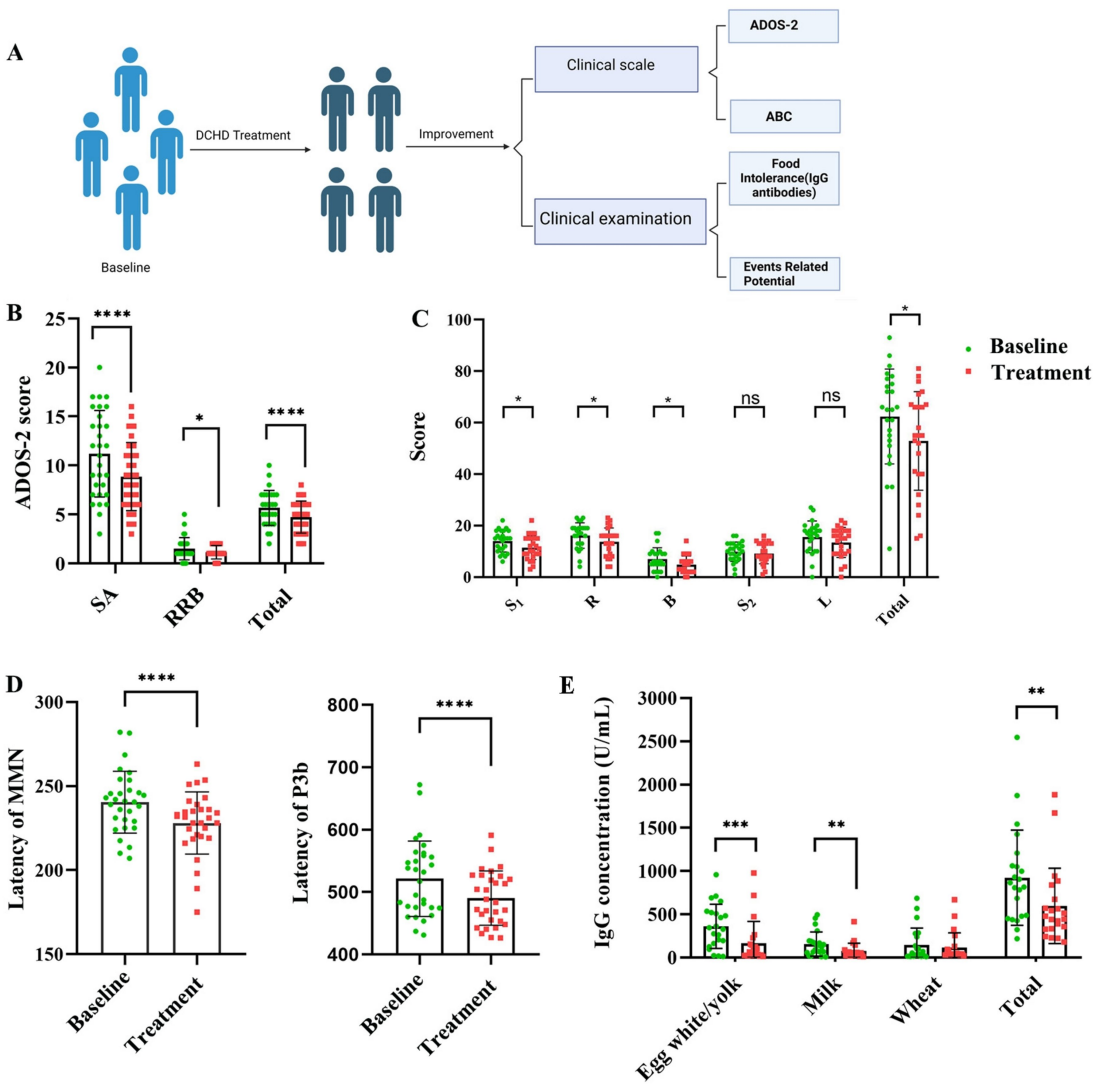
**(A)** Schematic of the small clinical study protocol. **(B,C)** DCHD effectively alleviates core ASD symptoms. **(D)** P3b latency and MMN latency, and **(E)** Food intolerance in children with ASD; *n* = 25–30, each group; social affect, SA; restricted and repetitive behavior, RRB. Two-sided Wilcoxon’s matched-pairs signed-rank test was used for multiple pairwise comparison. Sensory: S_1_; Relating: R; Body and object use: B; Social and selfhelp: S_2_; Language: L. *p < 0.05, **p < 0.01, ***p < 0.001, ****p < 0.0001, ns, not significant.

To explore the therapeutic effect of DCHD in children with ASD, we performed a small clinical study, revealing that 6 months of DCHD application as ASD treatment achieved better results (Figure 7), primarily evidenced by improved overall symptom severity as assessed by the ABC score and ADOS-2 (Figures 7B,C). Furthermore, the latencies of MMN and P3b were significantly reduced, suggesting the enhanced capacity to process social and emotional information upon treating ASD-affected children with DCHD (Figure 7D). Meanwhile, DCHD treatment significantly decreased the food intolerance by reducing the concentrations of total IgG antibodies and specifically the IgG antibodies of egg white/yolk and milk (Figure 7E). Notably, no adverse effects were observed in ASD-affected children treated with DCHD in this trial. DCHD had no effects on serum AST, ALT and *γ*-GT(liver function) and BUN and CREA(kidney function) (Supplementary Figure S4), suggesting its safety.

## 4 Discussion

ASD is one of the most common neurodevelopmental disorders in children, with a prevalence rate of >2% in western countries (Maenner et al., 2023). The gut microbiota could be a promising therapeutic target for the interventions in ASD. The TCM formula DCHD reportedly exerts regulatory effects on the gut microbiota, which may be beneficial in ASD. However, the specific efficacy and mechanisms of DCHD in treating ASD remain unclear. Herein, DCHD significantly improved ASD-like behaviors, decreased inflammation and oxidative stress, restored gut barrier function, and modulated the gut microbiota in a PPA-induced rat model of ASD. Moreover, the DCHD dose that achieved such improvements was safe and well tolerated; this was consistent with the findings of a previous study (Cui et al., 2020), suggesting that DCHD is safe and effective for treating ASD.

The gut–brain axis is a complex bidirectional communication system between the gastrointestinal tract and the central nervous system, which has been implicated in various neurological disorders, including ASD (Morton et al., 2023). Gut barrier dysfunction, characterized by increased tight junction permeability and loss of tight junction proteins, is common in ASD (Dargenio et al., 2023). The changes in epithelial barrier function induced by DCHD were investigated here. Decreased serum levels of DAO and DLA, along with increased protein expression of the gut -barrier associated marker ZO-1 and occludin in the colon, were observed in DCHD-treated rats. Moreover, compared with ASD rats, DCHD treatment decreased the number of microglia and arranged more tightly neurons in the DG region of the hippocampus, indicating the memory and learning deficits was partially restored (Liu et al., 2023). The results suggested that DCHD might improve gut barrier and hippocampal function, alleviating ASD symptoms via the gut–brain axis.

Given that ASD is inextricably linked to alterations in the gut microbiota, the crosstalk between DCHD and the gut microbiota was analyzed here. The 16S rRNA sequencing showed that DCH_H treatment significantly attenuated gut dysbiosis in autistic rats (Figure 4). DCH_H treatment reversed the changes in *Firmicutes*/*Bacteroidetes* ratio, which was reported to increase in an ASD rat model (Jingyi et al., 2024). Moreover, the decreases in the probiotics *Adlercreutzia* and *Christensenellaceae*_unclassified induced by PPA were significantly reversed by treatment with DCH_H. Additionally, other probiotics such as *Faecalibaculum*, *Dubosiella*, *Parvibacter*, *Romboutsia*, *Turicibacter*, and *Ruminococcaceae*_UCG-007 were enriched by the DCH_H treatment. *Adlercreutzia* has reportedly played an active role in controlling inflammation and protecting the intestinal barrier (Wang et al., 2024), and to be negatively correlated with anxiety-like behavior (Xu et al., 2019). Moreover, *Faecalibaculum*, *Romboutsia*, and *Ruminococcaceae* are reportedly associated with enhanced gut motility and gut epithelial barrier by producing SCFAs (Li et al., 2021; Li et al., 2019; Li et al., 2022). Furthermore, the anti-inflammatory bacteria *Parvibacter*, *Christensenellaceae*, and *Dubosiella* are negatively correlated with IL-6 and TNF-*α*, and positively correlated with IL-10 (Figure 6A), which is consistent with the findings in previous reports(Chen et al., 2024; Gao et al., 2018; Wan et al., 2021). In addition, the abundance of *Turicibacter* is decreased in ASD-affected children compared with that in typically developing controls (Mehra et al., 2023). Notably, DCHD treatment significantly increased the relative abundance of *Turicibacter*, a critical component of the gut microbiota that plays a potential role in gut health (Wang et al., 2023). Considerably, DCHD treatment increased beneficial microbiota and inhibited inflammation and intestinal barrier damage, potentially contributing to improved ASD-like social behaviors.

The systematicity of metabolomics is similar to the holistic nature of TCM (Wang et al., 2024). Indeed, upon its administration, TCM can cause changes in the gut microbiota metabolome. Herein, the metabolomic analysis revealed that numerous metabolites were upregulated in DCHD-treated rats, including indole-3-acetate, apigenin, erythritol, and N-acetyl-L-glutamine. Of these, apigenin (Jayaprakash et al., 2024), N-acetyl-L-glutamine (Wu et al., 2023), and indole-3-acetate (Wei et al., 2024) are known for their anti-inflammatory, antioxidant, and gut–barrier protective effects. Specifically, apigenin and erythritol alleviate autistic-like stereotyped repetitive behaviors by mitigating oxidative stress and curbing excessive proliferation of the gut microbiota in ASD model mice (Cannon et al., 2020; Soltani et al., 2024). N-Acetyl-L-glutamine possess neuroprotective effects in various nervous system disorders, such as hemiplegia, brain trauma, and cerebral apoplexy sequelae (Ding et al., 2015; Wu et al., 2023). Meanwhile, indole-3-acetate is reduced in fecal samples from children with ASD compared with the levels in control group (Peralta-Marzal et al., 2021). In addition, the present findings revealed that DCHD downregulates asymmetric dimethylarginine and homogentisic acid. These compounds are known to elevate oxidative stress, potentially increasing ROS generation and resulting in redox imbalance, which can cause cerebral endothelial dysfunction and contribute to cognitive disorders (Braconi et al., 2010; Braconi et al., 2015; Jiang et al., 2024). Our results indicate that alterations in the gut microbiota metabolome may contribute to the increased antioxidant ability and decreased inflammatory response, thereby improving the ASD-like symptoms observed in rats with PPA-induced ASD. The altered metabolites identified in the metabolomic analysis may serve as major players in the effectiveness of DCHD treatment. Our findings suggested possible crosstalk between DCHD and the gut microbiota metabolome improves social behaviors in autistic rats.

Integrative gut microbiome and metabolome analysis revealed that the anti-inflammatory and antioxidant bacteria *Adlercreutzia* and *Faecalibaculum* were positively correlated with apigenin and erythritol in our study. Such metabolism can reduce repetitive behaviors of mice with ASD. Another anti-inflammatory bacterial taxon, the genus *Parvibacter*, was negatively correlated with asymmetric dimethylarginine, a metabolite associated with exacerbating oxidative stress. In addition, the abundances of *Parvibacter*, *Rombo*utsia, and Christensenellaceae_uncultured are linked to improved social behaviors (Figure 6A). Herein, the abundances of *Adlercreutzia*, *Faecalibaculum*, *Parvibacter*, *Romboutsia*, and *Christensenellaceae*_uncultured were significantly increased by DCHD, suggesting that DCHD may improve abnormal behavior in mice with ASD by remodeling the gut microbiota and improving inflammatory responses. Thus, the protective role of DCHD in ASD involves modified gut microbiota and metabolites, highlighting the interplay between DCHD and gut microbiota in alleviating ASD symptoms. These findings may provide new insights into therapeutic interventions for ASD.

Our small clinical trial further demonstrated that DCHD significantly alleviated core symptoms of ASD, resulting in improvements in overall symptom severity, social communication, and motivation among children with ASD. DCHD also ameliorated gastrointestinal problems, as evidenced by decreased levels of FI-specific IgG antibody and total IgG antibody (Li et al., 2020). Additionally, the MMN (Chien et al., 2024) and P3b latencies (Zhou et al., 2023) were significantly shortened by DCHD treatment, indicating improved cognitive impairment and brain responses for discriminating novelty. Given the clinical data about children with ASD, DCHD treatment may be promising for improving the main symptoms of ASD without side effects. In future studies, we plan to adopt multisource targeted metabolomics, metagenomics, and FMT to explore the association between differences in gut microbiota variations and metabolite changes in ASD patients after DCHD administration.

## 5 Conclusion

Combining clinical data from children with ASD and findings from interventional experiments in rats, this study confirmed the safety and efficacy of DCHD for treating ASD. The mechanism of action could be associated with reducing oxidative stress and modulating inflammatory reactions, protecting the intestinal barrier, balancing gut microbiota, and modulating gut microbial metabolism. This study provided crucial directions for the discovery and advancement of therapeutic agents for treating ASD and other psychiatric conditions at the gut microbiota and metabolic levels.

## Data Availability

The datasets presented in this study can be found in online repositories. The names of the repository/repositories and accession number(s) can be found in the article/Supplementary material.

## References

[ref1] Abdelrazig S. Safo L. Rance G. A. Fay M. W. Theodosiou E. Topham P. D.. (2020). Metabolic characterisation of *Magnetospirillum gryphiswaldense* MSR-1 using LC-MS-based metabolite profiling. RSC Adv. 10, 32548–32560. doi: 10.1039/D0RA05326K, PMID: 35516490 PMC9056635

[ref2] Alexandrov P. N. Hill J. M. Zhao Y. Bond T. Taylor C. M. Percy M. E.. (2020). Aluminum-induced generation of lipopolysaccharide (LPS) from the human gastrointestinal (GI)-tract microbiome-resident *Bacteroides fragilis*. J. Inorg. Biochem. 203:110886. doi: 10.1016/j.jinorgbio.2019.110886, PMID: 31707334 PMC7409391

[ref3] Banker S. M. Gu X. Schiller D. Foss-Feig J. H. (2021). Hippocampal contributions to social and cognitive deficits in autism spectrum disorder. Trends Neurosci. 44, 793–807. doi: 10.1016/j.tins.2021.08.005, PMID: 34521563 PMC8484056

[ref4] Benitah K. C. Kavaliers M. Ossenkopp K. P. (2023). The enteric metabolite, propionic acid, impairs social behavior and increases anxiety in a rodent ASD model: examining sex differences and the influence of the estrous cycle. Pharmacol. Biochem. Behav. 231:173630. doi: 10.1016/j.pbb.2023.173630, PMID: 37640163

[ref5] Biswas M. Vanwong N. Sukasem C. (2023). Pharmacogenomics and non-genetic factors affecting drug response in autism spectrum disorder in Thai and other populations: current evidence and future implications. Front. Pharmacol. 14:1285967. doi: 10.3389/fphar.2023.1285967 38375208 PMC10875059

[ref6] Bougeard C. Picarel-Blanchot F. Schmid R. Campbell R. Buitelaar J. (2024). Prevalence of autism Spectrum disorder and co-morbidities in children and adolescents: a systematic literature review. Focus (Am Psychiatr Publ) 22, 212–228. doi: 10.1176/appi.focus.24022005, PMID: 38680973 PMC11046711

[ref7] Braconi D. Laschi M. Taylor A. M. Bernardini G. Spreafico A. Tinti L.. (2010). Proteomic and redox-proteomic evaluation of homogentisic acid and ascorbic acid effects on human articular chondrocytes. J. Cell. Biochem. 111, 922–932. doi: 10.1002/jcb.22780, PMID: 20665660

[ref8] Braconi D. Millucci L. Bernardini G. Santucci A. (2015). Oxidative stress and mechanisms of ochronosis in alkaptonuria. Free Radic. Biol. Med. 88, 70–80. doi: 10.1016/j.freeradbiomed.2015.02.021, PMID: 25733348

[ref9] Buffington S. A. Di Prisco G. V. Auchtung T. A. Ajami N. J. Petrosino J. F. Costa-Mattioli M. (2016). Microbial reconstitution reverses maternal diet-induced social and synaptic deficits in offspring. Cell 165, 1762–1775. doi: 10.1016/j.cell.2016.06.001, PMID: 27315483 PMC5102250

[ref10] Cannon M. L. Merchant M. Kabat W. Unruh B. Ramones A. (2020). Inhibition of autism Spectrum disorder associated Bacteria and *C. difficile* by polyols. Edelweiss applied. Sci. Technol. 33-36, 33–36. doi: 10.33805/2576-8484.176

[ref11] Chelakkot C. Ghim J. Ryu S. H. (2018). Mechanisms regulating intestinal barrier integrity and its pathological implications. Exp. Mol. Med. 50, 1–9. doi: 10.1038/s12276-018-0126-x, PMID: 30115904 PMC6095905

[ref12] Chen Y. C. Lin H. Y. Chien Y. Tung Y. H. Ni Y. H. Gau S. S. (2022). Altered gut microbiota correlates with behavioral problems but not gastrointestinal symptoms in individuals with autism. Brain Behav. Immun. 106, 161–178. doi: 10.1016/j.bbi.2022.08.015, PMID: 36058421

[ref13] Chen D. Wang Y. Yang J. Ou W. Lin G. Zeng Z.. (2024). Shenling Baizhu san ameliorates non-alcoholic fatty liver disease in mice by modulating gut microbiota and metabolites. Front. Pharmacol. 15:1343755. doi: 10.3389/fphar.2024.1343755, PMID: 38720776 PMC11076757

[ref14] Chien Y. L. Hsieh M. H. Gau S. S. (2024). Mismatch negativity and P3a in unaffected siblings of individuals with autism Spectrum disorder and the exploration on the neurocognitive implications. J. Autism Dev. Disord. 2024:520. doi: 10.1007/s10803-024-06520-1, PMID: 39242471

[ref15] Cui H. Li Y. Wang Y. Jin L. Yang L. Wang L.. (2020). Da-chai-Hu decoction ameliorates high fat diet-induced nonalcoholic fatty liver disease through remodeling the gut microbiota and modulating the serum metabolism. Front. Pharmacol. 11:584090. doi: 10.3389/fphar.2020.584090, PMID: 33328987 PMC7732620

[ref16] Dargenio V. N. Dargenio C. Castellaneta S. De Giacomo A. Laguardia M. Schettini F.. (2023). Intestinal barrier dysfunction and microbiota-gut-brain Axis: possible implications in the pathogenesis and treatment of autism Spectrum disorder. Nutrients 15:620. doi: 10.3390/nu15071620, PMID: 37049461 PMC10096948

[ref17] Ding L. Dong G. Zhang D. Ni Y. Hou Y. (2015). The regional function of cGAS/STING signal in multiple organs: one of culprit behind systemic lupus erythematosus? Med. Hypotheses 85, 846–849. doi: 10.1016/j.mehy.2015.09.026, PMID: 26464144

[ref18] Duan Z. W. Liu Y. Zhang P. P. Hu J. Y. Mo Z. X. Liu W. Q.. (2024). Da-chai-Hu-Tang formula inhibits the progression and metastasis in HepG2 cells through modulation of the PI3K/AKT/STAT3-induced cell cycle arrest and apoptosis. J. Ethnopharmacol. 331:118293. doi: 10.1016/j.jep.2024.118293, PMID: 38705430

[ref19] Fischbach M. A. Segre J. A. (2016). Signaling in host-associated microbial communities. Cell 164, 1288–1300. doi: 10.1016/j.cell.2016.02.037, PMID: 26967294 PMC4801507

[ref20] Gao L. Li J. Zhou Y. Huang X. Qin X. Du G. (2018). Effects of Baicalein on cortical Proinflammatory cytokines and the intestinal microbiome in senescence accelerated mouse prone 8. ACS Chem. Neurosci. 9, 1714–1724. doi: 10.1021/acschemneuro.8b00074, PMID: 29668250

[ref21] Guidetti C. Salvini E. Viri M. Deidda F. Amoruso A. Visciglia A.. (2022). Randomized double-blind crossover study for evaluating a probiotic mixture on gastrointestinal and behavioral symptoms of autistic children. J. Clin. Med. 11:263. doi: 10.3390/jcm11185263, PMID: 36142909 PMC9504504

[ref22] Horai H. Arita M. Kanaya S. Nihei Y. Ikeda T. Suwa K.. (2010). MassBank: a public repository for sharing mass spectral data for life sciences. J. Mass Spectrom. 45, 703–714. doi: 10.1002/jms.1777, PMID: 20623627

[ref23] Huang N. Wei Y. Liu M. Yang Z. Yuan K. Chen J.. (2023). Dachaihu decoction ameliorates septic intestinal injury via modulating the gut microbiota and glutathione metabolism as revealed by multi-omics. J. Ethnopharmacol. 312:116505. doi: 10.1016/j.jep.2023.116505, PMID: 37080366

[ref24] Jayaprakash P. Isaev D. Yang K. S. Beiram R. Oz M. Sadek B. (2024). Apigenin alleviates autistic-like stereotyped repetitive behaviors and mitigates brain oxidative stress in mice. Pharmaceuticals (Basel) 17:482. doi: 10.3390/ph17040482, PMID: 38675442 PMC11054933

[ref25] Jiang X. Chen X. Su J. Liu N. (2024). Prevalence of autism spectrum disorder in mainland China over the past 6 years: a systematic review and meta-analysis. BMC Psychiatry 24:404. doi: 10.1186/s12888-024-05729-9, PMID: 38811881 PMC11137880

[ref26] Jiang J. Miao W. Guo H. Tian W. (2024). Effects of rosuvastatin on serum asymmetric dimethylarginine levels and incidence of long-term cardiovascular events in patients with hyperlipidaemia and H-type hypertension. Br. J. Hosp. Med. (Lond.) 85, 1–10. doi: 10.12968/hmed.2024.0115, PMID: 39078896

[ref27] Jingyi L. Lin W. Yuan C. Lingling Z. Qianqian J. Anlong X.. (2024). Intravenous transplantation of bone marrow-derived mesenchymal stem cells improved behavioral deficits and altered fecal microbiota composition of BTBR mice. Life Sci. 336:122330. doi: 10.1016/j.lfs.2023.122330, PMID: 38065352

[ref28] Kang D.-W. Adams J. B. Gregory A. C. Borody T. Chittick L. Fasano A.. (2017). Microbiota transfer therapy alters gut ecosystem and improves gastrointestinal and autism symptoms: an open-label study. Microbiome 5:10. doi: 10.1186/s40168-016-0225-7, PMID: 28122648 PMC5264285

[ref29] Kelly J. R. Kennedy P. J. Cryan J. F. Dinan T. G. Clarke G. Hyland N. P. (2015). Breaking down the barriers: the gut microbiome, intestinal permeability and stress-related psychiatric disorders. Front. Cell. Neurosci. 9:392. doi: 10.3389/fncel.2015.00392, PMID: 26528128 PMC4604320

[ref30] Kraeuter A. K. Guest P. C. Sarnyai Z. (2019). The open field test for measuring locomotor activity and anxiety-like behavior. Methods Mol. Biol. 1916, 99–103. doi: 10.1007/978-1-4939-8994-2_9 30535687

[ref31] Li N. Chen H. Cheng Y. Xu F. Ruan G. Ying S.. (2021). Fecal microbiota transplantation relieves gastrointestinal and autism symptoms by improving the gut microbiota in an open-label study. Frontiers in cellular and infection. Microbiology 11:376. doi: 10.3389/fcimb.2021.801376, PMID: 34737978 PMC8560686

[ref32] Li J. W. Fang B. Pang G. F. Zhang M. Ren F. Z. (2019). Age- and diet-specific effects of chronic exposure to chlorpyrifos on hormones, inflammation and gut microbiota in rats. Pestic. Biochem. Physiol. 159, 68–79. doi: 10.1016/j.pestbp.2019.05.018, PMID: 31400786

[ref33] Li G. Lin J. Zhang C. Gao H. Lu H. Gao X.. (2021). Microbiota metabolite butyrate constrains neutrophil functions and ameliorates mucosal inflammation in inflammatory bowel disease. Gut Microbes 13:1968257. doi: 10.1080/19490976.2021.1968257, PMID: 34494943 PMC8437544

[ref34] Li C. Liu Y. Fang H. Chen Y. Weng J. Zhai M.. (2020). Study on aberrant eating behaviors, food intolerance, and stereotyped behaviors in autism Spectrum disorder. Front. Psych. 11:493695. doi: 10.3389/fpsyt.2020.493695, PMID: 33240114 PMC7678488

[ref35] Li Y. Xia D. Chen J. Zhang X. Wang H. Huang L.. (2022). Dietary fibers with different viscosity regulate lipid metabolism via ampk pathway: roles of gut microbiota and short-chain fatty acid. Poult. Sci. 101:101742. doi: 10.1016/j.psj.2022.101742, PMID: 35245807 PMC8892021

[ref36] Lin C. H. Zeng T. Lu C.-W. Li D.-Y. Liu Y.-Y. Li B.-M.. (2024). Efficacy and safety of *Bacteroides fragilis* BF839 for pediatric autism spectrum disorder: a randomized clinical trial. Front. Nutr. 11:59. doi: 10.3389/fnut.2024.1447059, PMID: 39290561 PMC11407114

[ref37] Liu Y. Li H. Wang X. Huang J. Zhao D. Tan Y.. (2023). Anti-Alzheimers molecular mechanism of icariin: insights from gut microbiota, metabolomics, and network pharmacology. J. Transl. Med. 21:277. doi: 10.1186/s12967-023-04137-z, PMID: 37095548 PMC10124026

[ref38] Liu Q. Q. Mi J. Du Y. Y. Rong Z. Qin Y. Jiang W.. (2024). Lotusine ameliorates propionic acid-induced autism spectrum disorder-like behavior in mice by activating D1 dopamine receptor in medial prefrontal cortex. Phytother. Res. 38, 1089–1103. doi: 10.1002/ptr.8098, PMID: 38168755

[ref39] Ma Y. Z. Zhang Y. S. Cao J. X. Chen H. C. Su X. M. Li B.. (2024). Aberration of social behavior and gut microbiota induced by cross-fostering implicating the gut-brain axis. Brain Behav. Immun. 120, 499–512. doi: 10.1016/j.bbi.2024.06.026, PMID: 38944162

[ref40] Maenner M. J. Warren Z. Williams A. R. Amoakohene E. Bakian A. V. Bilder D. A.. (2023). Prevalence and characteristics of autism Spectrum disorder among children aged 8 years - autism and developmental disabilities monitoring network, 11 sites, United States, 2020. Morbidity and mortality weekly report. MMWR Surveill Summ 72, 1–14. doi: 10.15585/mmwr.ss7202a1 PMC10042614 36952288

[ref41] Mazzone L. Dooling S. W. Volpe E. Uljarević M. Waters J. L. Sabatini A.. (2024). Precision microbial intervention improves social behavior but not autism severity: a pilot double-blind randomized placebo-controlled trial. Cell Host Microbe 32, 106–116.e6. doi: 10.1016/j.chom.2023.11.021, PMID: 38113884

[ref42] Mehra A. Arora G. Sahni G. Kaur M. Singh H. Singh B.. (2023). Gut microbiota and autism Spectrum disorder: from pathogenesis to potential therapeutic perspectives. J. Tradit. Complement. Med. 13, 135–149. doi: 10.1016/j.jtcme.2022.03.001, PMID: 36970459 PMC10037072

[ref43] Morton J. T. Jin D. M. Mills R. H. Shao Y. Rahman G. McDonald D.. (2023). Multi-level analysis of the gut-brain axis shows autism spectrum disorder-associated molecular and microbial profiles. Nat. Neurosci. 26, 1208–1217. doi: 10.1038/s41593-023-01361-0, PMID: 37365313 PMC10322709

[ref44] Ogata H. Goto S. Sato K. Fujibuchi W. Bono H. Kanehisa M. (1999). KEGG: Kyoto encyclopedia of genes and genomes. Nucleic Acids Res. 27, 29–34. doi: 10.1093/nar/27.1.29, PMID: 9847135 PMC148090

[ref45] Peralta-Marzal L. N. Prince N. Bajic D. Roussin L. Naudon L. Rabot S.. (2021). The impact of gut microbiota-derived metabolites in autism Spectrum disorders. Int. J. Mol. Sci. 22:52. doi: 10.3390/ijms221810052, PMID: 34576216 PMC8470471

[ref46] Poupard L. Page G. Thoreau V. Kaouah Z. (2024). Relationships between gut microbiota and autism Spectrum disorders: development and treatment. Clin Psychopharmacol Neurosci 22, 554–564. doi: 10.9758/cpn.24.1179, PMID: 39420603 PMC11494427

[ref47] Qin L. Wang H. Ning W. Cui M. Wang Q. (2024). New advances in the diagnosis and treatment of autism spectrum disorders. Eur. J. Med. Res. 29:322. doi: 10.1186/s40001-024-01916-2, PMID: 38858682 PMC11163702

[ref48] Sherwin E. Bordenstein S. R. Quinn J. L. Dinan T. G. Cryan J. F. (2019). Microbiota and the social brain. Science 366:2016. doi: 10.1126/science.aar2016, PMID: 31672864

[ref49] Shi H. Sun M. Wang S. He F. Yang R. Li Z.. (2024). Jiawei Dachaihu decoction protects against mitochondrial dysfunction in atherosclerosis (AS) mice with chronic unpredictable mild stress (CUMS) via SIRT1/PGC-1alpha/TFAM/LON signaling pathway. J. Ethnopharmacol. 330:118150. doi: 10.1016/j.jep.2024.118150, PMID: 38631487

[ref50] Soltani Z. Shariatpanahi M. Aghsami M. Owliaey H. Kheradmand A. (2024). Investigating the effect of exposure to monosodium glutamate during pregnancy on development of autism in male rat offspring. Food Chem. Toxicol. 185:114464. doi: 10.1016/j.fct.2024.114464, PMID: 38244665

[ref51] Sud M. Fahy E. Cotter D. Brown A. Dennis E. A. Glass C. K.. (2007). LMSD: LIPID MAPS structure database. Nucleic Acids Res. 35, D527–D532. doi: 10.1093/nar/gkl838, PMID: 17098933 PMC1669719

[ref52] Tan Q. Orsso C. E. Deehan E. C. Kung J. Y. Tun H. M. Wine E.. (2021). Probiotics, prebiotics, synbiotics, and fecal microbiota transplantation in the treatment of behavioral symptoms of autism spectrum disorder: a systematic review. Autism Res. 14, 1820–1836. doi: 10.1002/aur.2560, PMID: 34173726

[ref53] Wan F. Han H. Zhong R. Wang M. Tang S. Zhang S.. (2021). Dihydroquercetin supplement alleviates colonic inflammation potentially through improved gut microbiota community in mice. Food Funct. 12, 11420–11434. doi: 10.1039/D1FO01422F, PMID: 34673859

[ref54] Wan L. Wang H. Liang Y. Zhang X. Yao X. Zhu G.. (2024). Effect of oral faecal microbiota transplantation intervention for children with autism spectrum disorder: a randomised, double-blind, placebo-controlled trial. Clin. Transl. Med. 14:e70006. doi: 10.1002/ctm2.70006, PMID: 39187939 PMC11347384

[ref55] Wang J. Cao Y. Hou W. Bi D. Yin F. Gao Y.. (2023). Fecal microbiota transplantation improves VPA-induced ASD mice by modulating the serotonergic and glutamatergic synapse signaling pathways. Transl. Psychiatry 13:17. doi: 10.1038/s41398-023-02307-7, PMID: 36670104 PMC9859809

[ref56] Wang N. Chen S. Xie Y. Liu X. Xi Z. Li J.. (2024). The Sanbi decoction alleviates intervertebral disc degeneration in rats through intestinal flora and serum metabolic homeostasis modulation. Phytomedicine 127:155480. doi: 10.1016/j.phymed.2024.155480 38484462

[ref57] Wei W. Liu Y. Hou Y. Cao S. Chen Z. Zhang Y.. (2024). Psychological stress-induced microbial metabolite indole-3-acetate disrupts intestinal cell lineage commitment. Cell Metab. 36, 466–483.e7. doi: 10.1016/j.cmet.2023.12.026, PMID: 38266651

[ref58] Wishart D. S. Tzur D. Knox C. Eisner R. Guo A. C. Young N.. (2007). HMDB: the human metabolome database. Nucleic Acids Res. 35, D521–D526. doi: 10.1093/nar/gkl923, PMID: 17202168 PMC1899095

[ref59] Wu L. Chen S. He B. Zhou G. Xu Y. Zhu G.. (2023). Acetylglutamine facilitates motor recovery and alleviates neuropathic pain after brachial plexus root avulsion in rats. J. Transl. Med. 21:563. doi: 10.1186/s12967-023-04399-7, PMID: 37612586 PMC10464467

[ref60] Xiao W. Su J. Gao X. Yang H. Weng R. Ni W.. (2022). The microbiota-gut-brain axis participates in chronic cerebral hypoperfusion by disrupting the metabolism of short-chain fatty acids. Microbiome 10:62. doi: 10.1186/s40168-022-01255-6, PMID: 35430804 PMC9013454

[ref61] Xu Z. Wang C. Dong X. Hu T. Wang L. Zhao W.. (2019). Chronic alcohol exposure induced gut microbiota dysbiosis and its correlations with neuropsychic behaviors and brain BDNF/Gabra1 changes in mice. Biofactors 45, 187–199. doi: 10.1002/biof.1469, PMID: 30417952

[ref62] Zarimeidani F. Rahmati R. Mostafavi M. Darvishi M. Khodadadi S. Mohammadi M.. (2024). Gut microbiota and autism Spectrum disorder: a Neuroinflammatory mediated mechanism of pathogenesis? Inflammation 2024:2061. doi: 10.1007/s10753-024-02061-y, PMID: 39093342 PMC12053372

[ref63] Zhang J. Zhu G. Wan L. Liang Y. Liu X. Yan H.. (2023). Effect of fecal microbiota transplantation in children with autism spectrum disorder: a systematic review. Front. Psych. 14:1123658. doi: 10.3389/fpsyt.2023.1123658, PMID: 36937721 PMC10017995

[ref64] Zheng H. Xu P. Jiang Q. Xu Q. Zheng Y. Yan J.. (2021). Depletion of acetate-producing bacteria from the gut microbiota facilitates cognitive impairment through the gut-brain neural mechanism in diabetic mice. Microbiome 9:145. doi: 10.1186/s40168-021-01088-9, PMID: 34172092 PMC8235853

[ref65] Zhou X. Lin Z. Yang W. Xiang M. Zhou B. Zou Z. (2023). The differences of event-related potential components in patients with comorbid depression and anxiety, depression, or anxiety alone. J. Affect. Disord. 340, 516–522. doi: 10.1016/j.jad.2023.08.049, PMID: 37572703

