# Supplementary figures and images for: Dachaihu decoction ameliorates abnormal behavior by regulating gut microbiota in rats with propionic acid-induced autism
Source: Front Microbiol. 2025 Feb 13;16:1535451. doi: 10.3389/fmicb.2025.1535451 (PMC11867326; doi:10.3389/fmicb.2025.1535451)

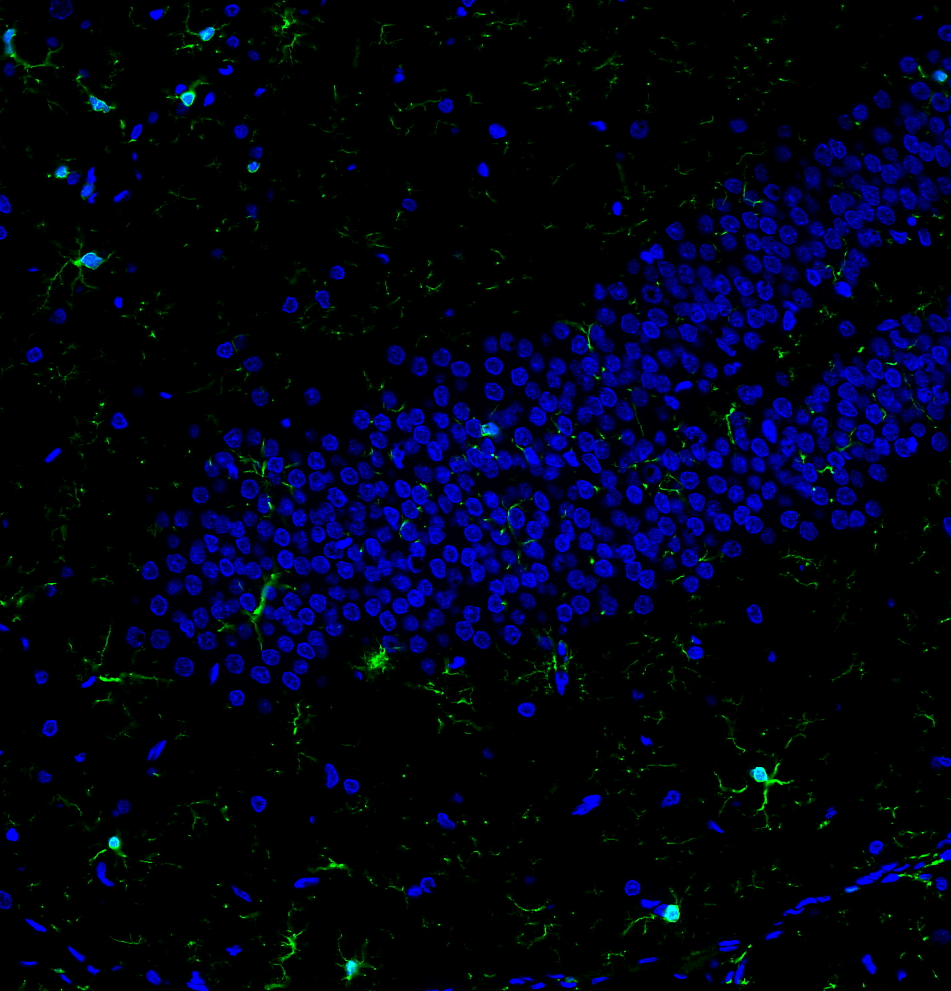

Supplement: Supplementary file 1 [file Data_Sheet_1.zip › Raw data/Original images/immunofluorescence/11 Green 200x.tiff]

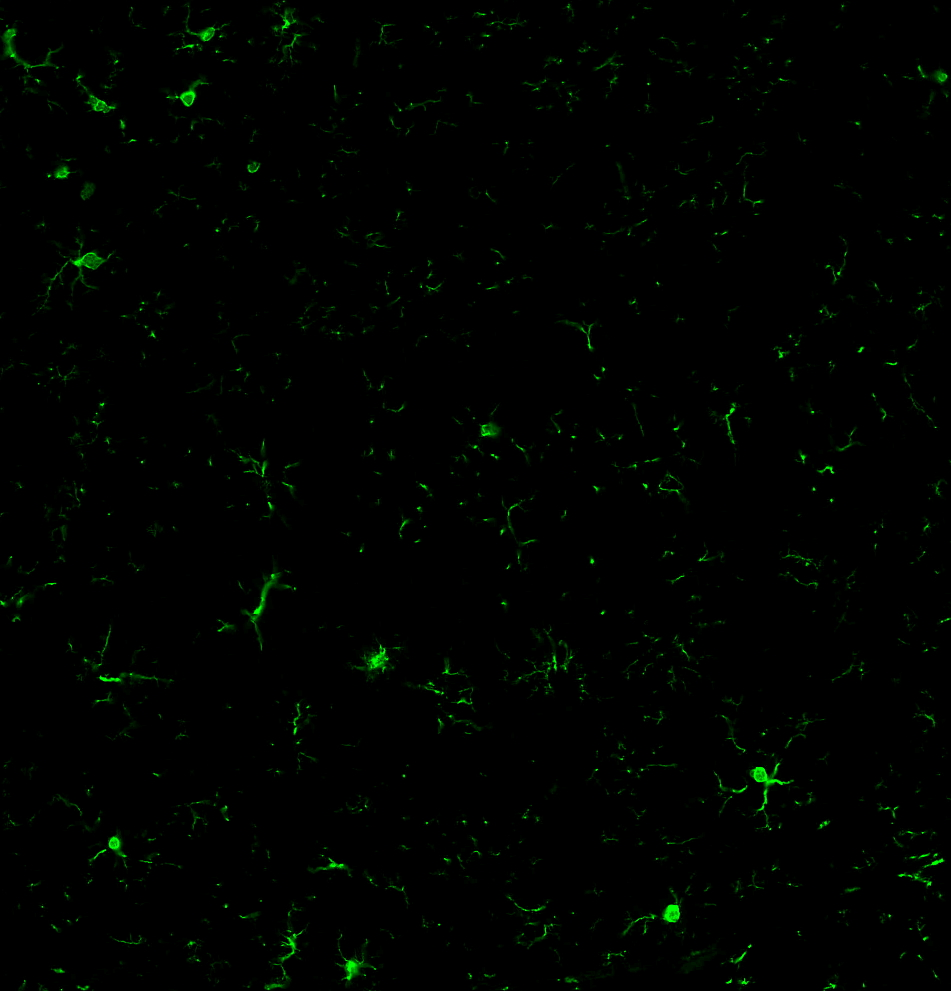

Supplement: Supplementary file 1 [file Data_Sheet_1.zip › Raw data/Original images/immunofluorescence/11Merge 200x.tiff]

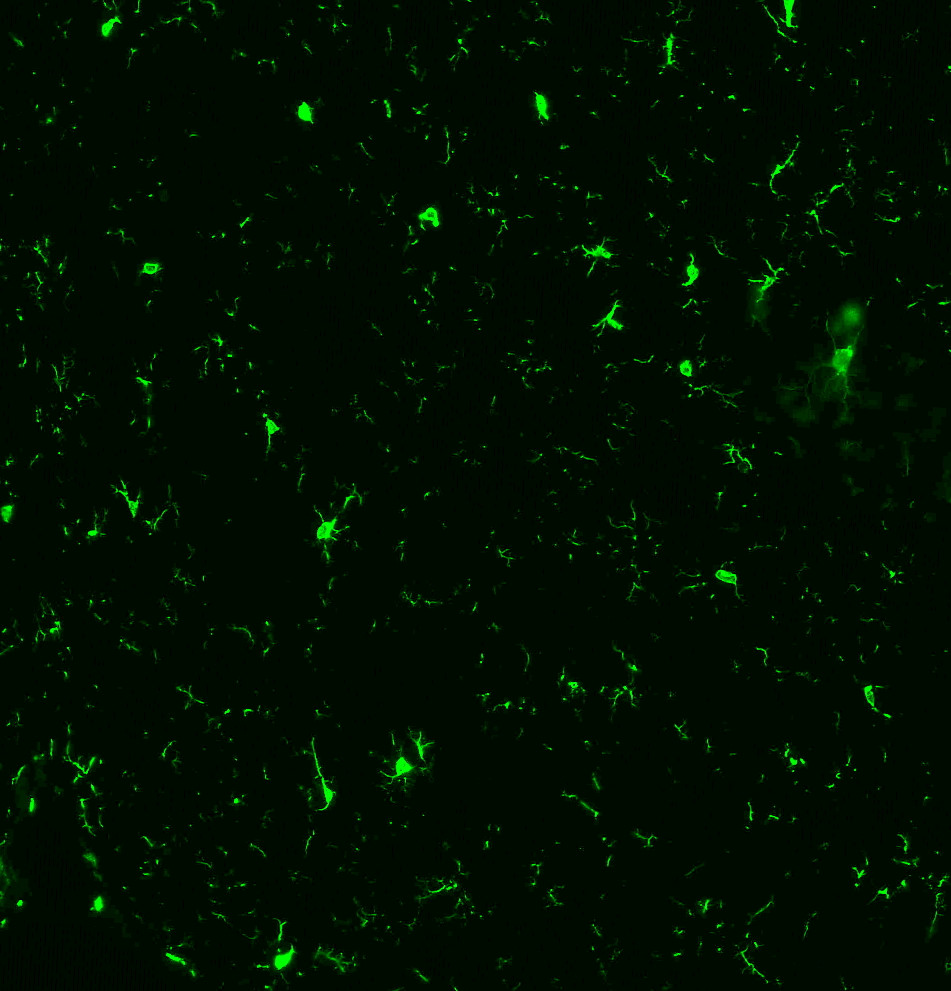

Supplement: Supplementary file 1 [file Data_Sheet_1.zip › Raw data/Original images/immunofluorescence/ASD Green 200x.tiff]

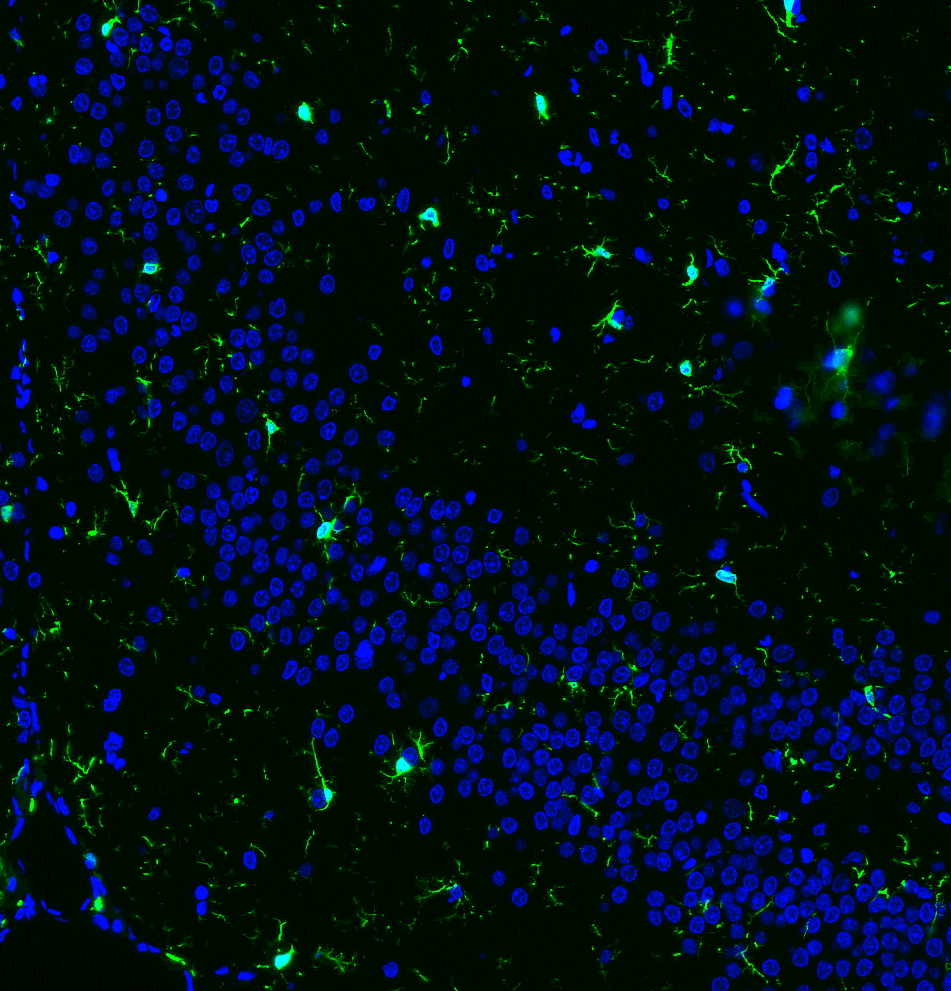

Supplement: Supplementary file 1 [file Data_Sheet_1.zip › Raw data/Original images/immunofluorescence/ASD Merge 200x.tiff]

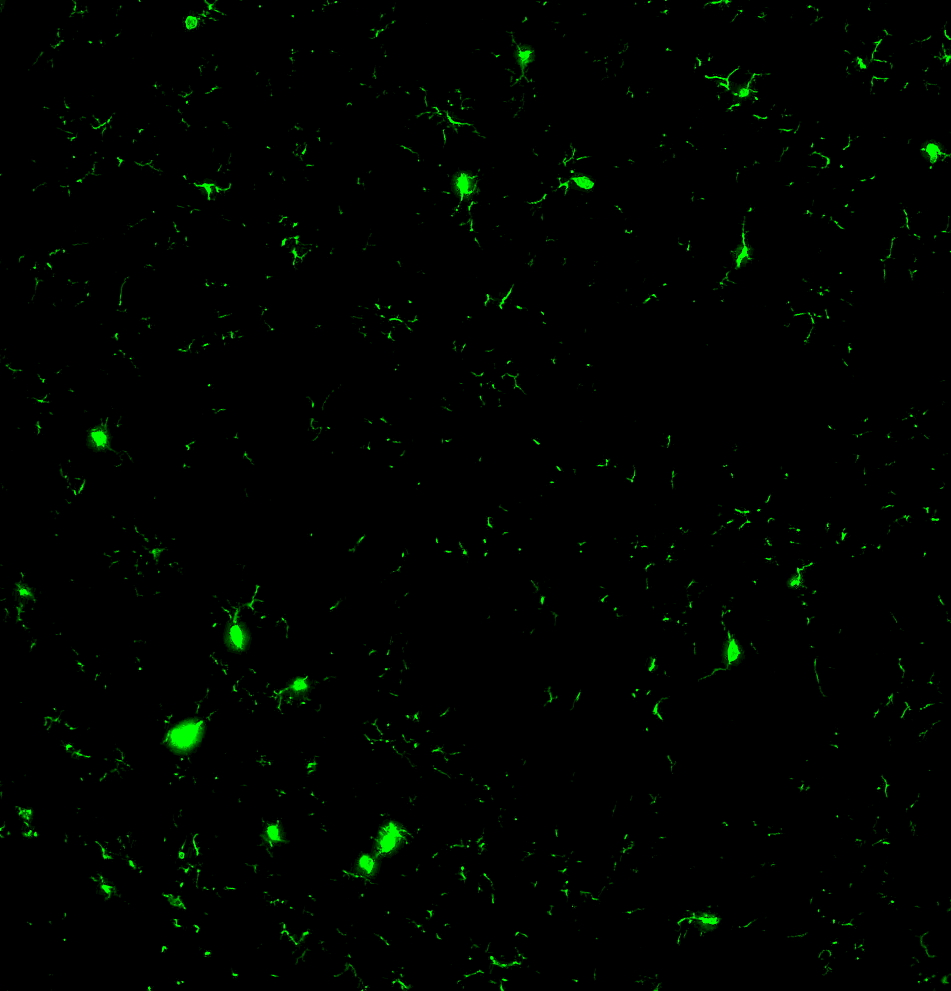

Supplement: Supplementary file 1 [file Data_Sheet_1.zip › Raw data/Original images/immunofluorescence/DCH_H+ASD Green 200x.tiff]

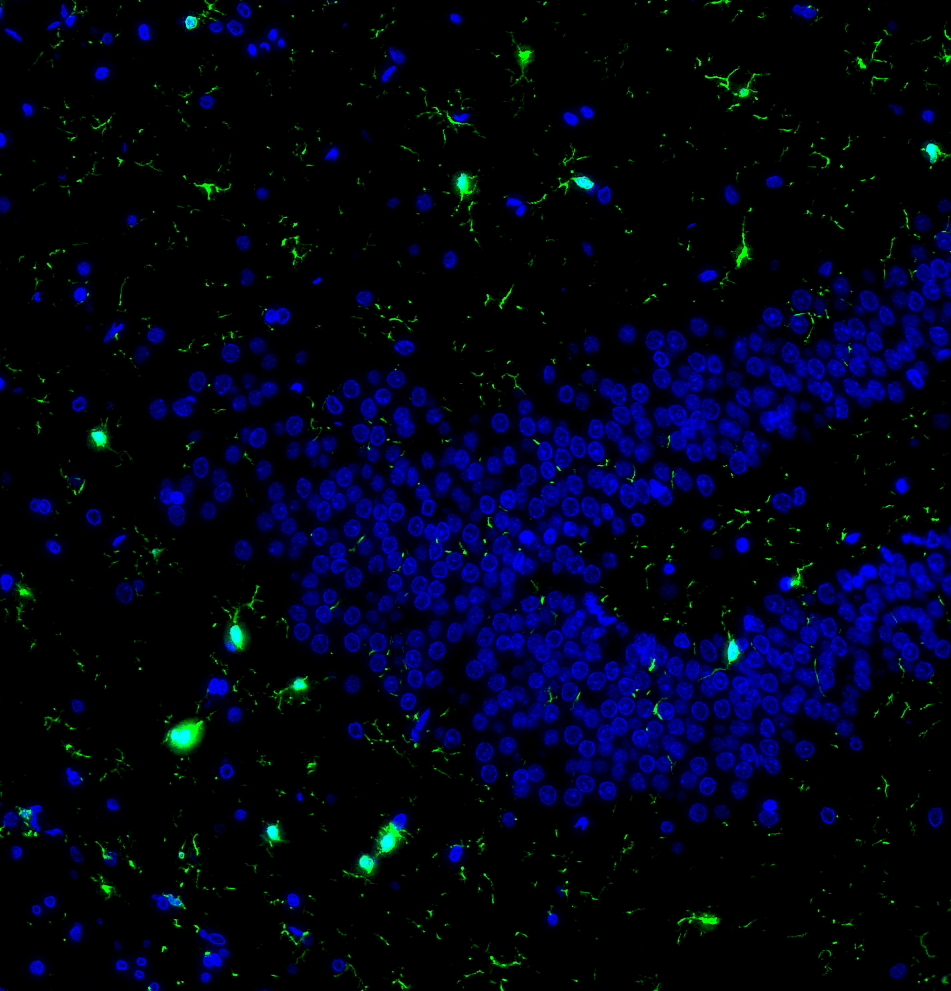

Supplement: Supplementary file 1 [file Data_Sheet_1.zip › Raw data/Original images/immunofluorescence/DCH_H+ASD Merge 200x.tiff]

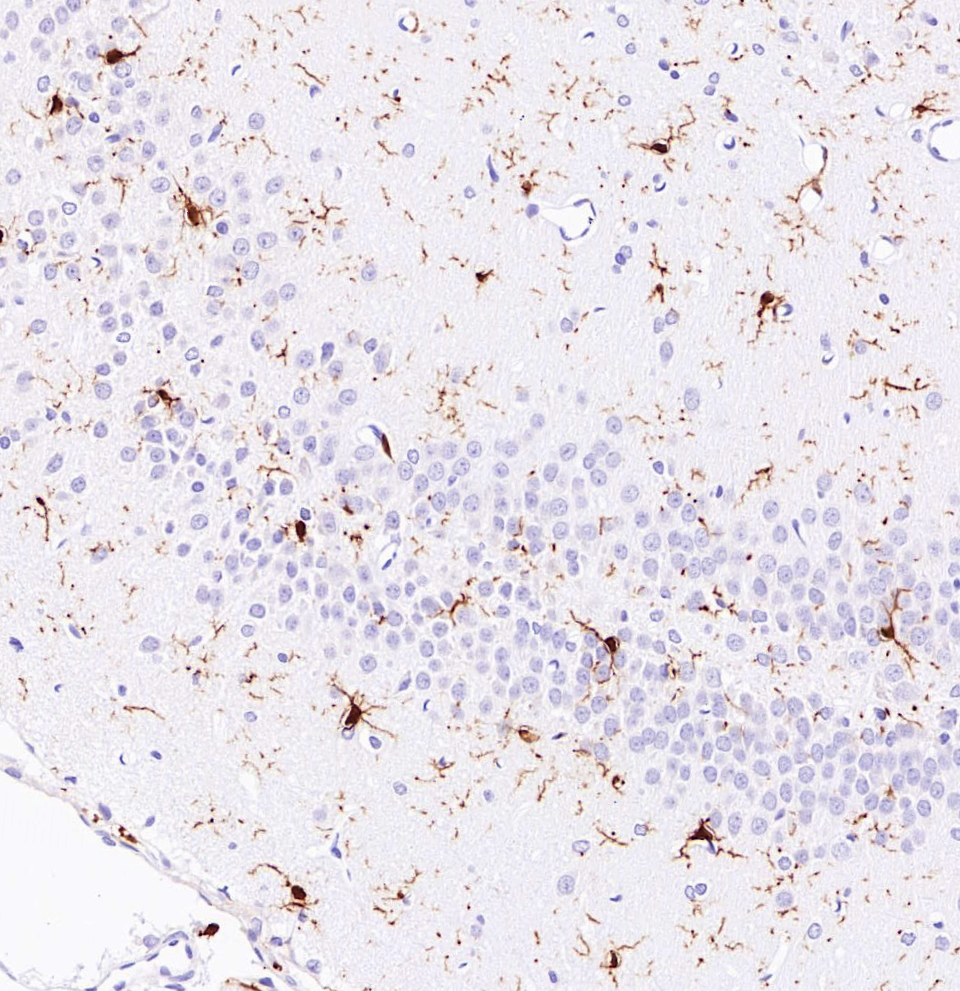

Supplement: Supplementary file 1 [file Data_Sheet_1.zip › Raw data/Original images/immunohistochemistry/ASD_200x.tiff]

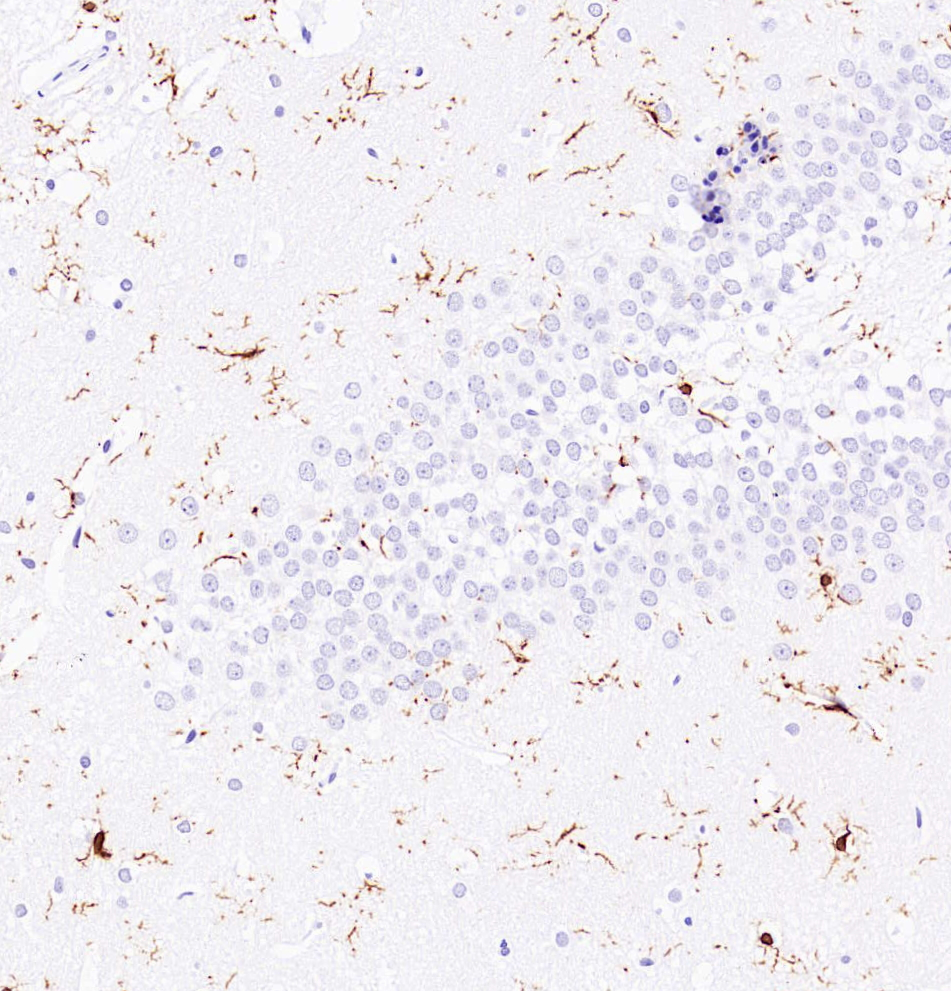

Supplement: Supplementary file 1 [file Data_Sheet_1.zip › Raw data/Original images/immunohistochemistry/Con_200x.tiff]

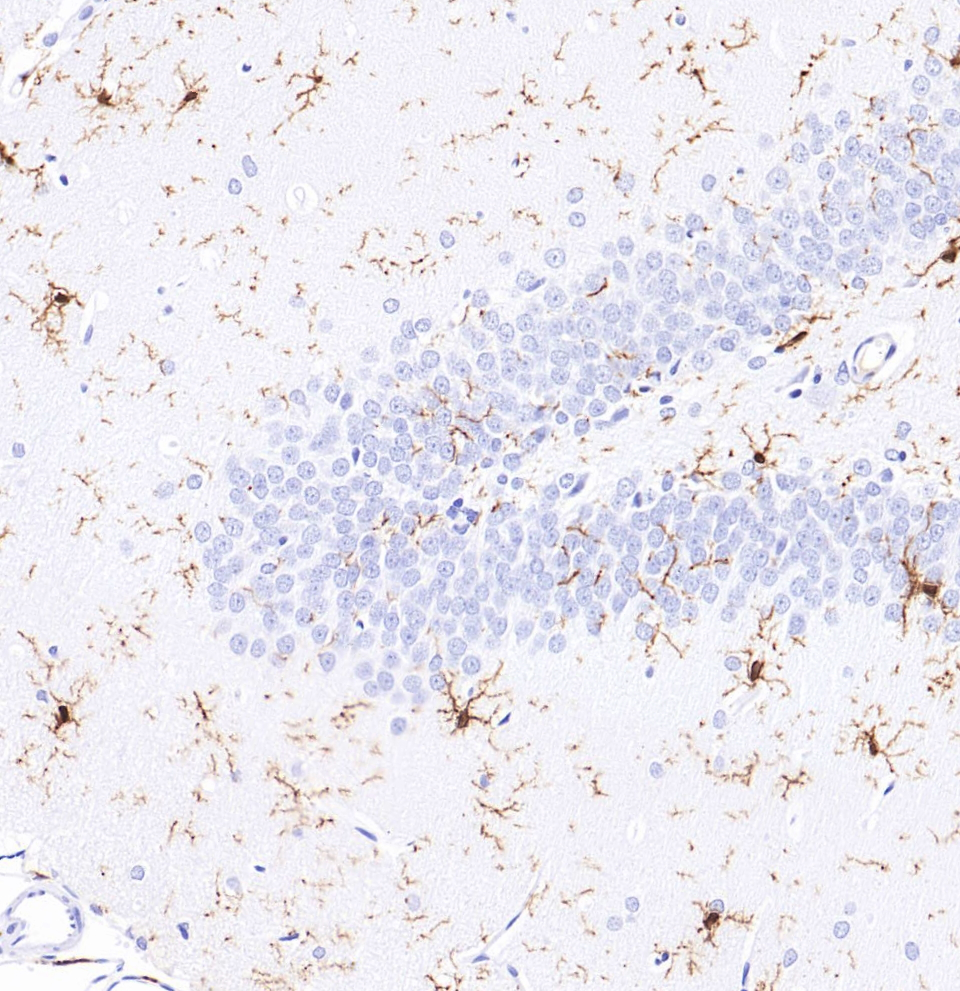

Supplement: Supplementary file 1 [file Data_Sheet_1.zip › Raw data/Original images/immunohistochemistry/DCH_H+ASD_200x.tiff]
