# Supplementary material for: Dachaihu decoction ameliorates abnormal behavior by regulating gut microbiota in rats with propionic acid-induced autism
Source: Front Microbiol. 2025 Feb 13;16:1535451. doi: 10.3389/fmicb.2025.1535451 (PMC11867326; doi:10.3389/fmicb.2025.1535451)
Supplement: Supplementary file 2 [file Supplementary_file_1.docx]

**Supplementary Materials**

***Dachaihu*** **decoction** **ameliorates abnormal behavior by regulating gut microbiota in rats with propionic acid-induced autism**

Yangyang Zhang^1^^#^, Hang Li^2#^, Bolin Li^3^, Yizhuang Li^4^, Xuejun Chai^5^, Sheng Li^6^,

Xia Xue^2^, Honglei Li^1^, Yonghong Zhao^1^, Youcai Tang^1,7^, Baoqi Yin^1^, Pengju Zhao^1^, Enyao Li^1*^, Pengya Feng^1,2*^

^1^ Department of Children Rehabilitation, Henan Key Laboratory of Rehabilitation Medicine, The Fifth Affiliated Hospital of Zhengzhou University, Zhengzhou, Henan, China

^2^ Henan Key Laboratory for Helicobacter pylori and Digestive Tract Microecology, The Fifth Affiliated Hospital of Zhengzhou University, Zhengzhou, China

^3^ College of Traditional Chinese Medicine, Fujian University of Traditional Chinese Medicine, Fuzhou, China

^4^ Department of Neurology, The Fifth Affiliated Hospital of Zhengzhou University, Zhengzhou, China

^5^ School of Basic Medical Science, The Shaanxi Key Laboratory of Brain Disorders, Xi'an Medical University, Xi'an, China

^6^ School of Life Sciences, Westlake University, Hangzhou, China.

^7^ Henan Provincial Outstanding Overseas Scientists Chronic Liver Injury Workshop; Zhengzhou Key Laboratory of Metabolism-Associated Fatty Liver Disease, The Fifth Affiliated Hospital of Zhengzhou University, Zhengzhou, China

*Corresponding author:

Enyao Li, Lienyao@zzu.edu.cn and Pengya Feng, [fengpy@zzu.edu.cn](mailto:fengpy@zzu.edu.cn,).

^#^ Yangyang Zhang and Hang Li contributed equally to this study


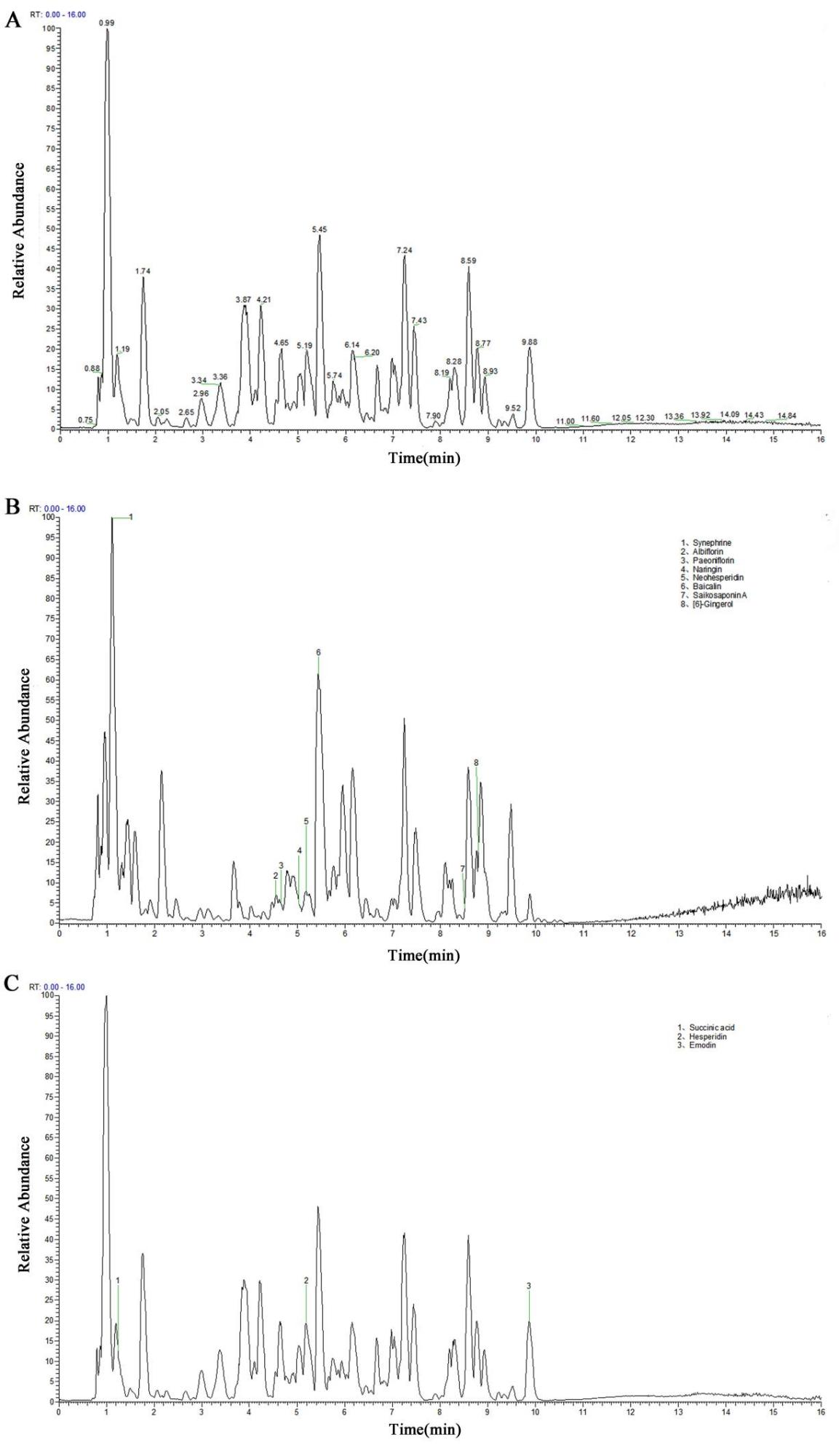


**Fig. S1 Ultrahigh-performance liquid chromatography–mass spectrometry of the main** active **ingredients in DCHD. A.** Total ion chromatograms of DCHD were obtained in positive- and negative-ion modes. **B.** Representative ion chromatograms of the eight components of DCHD obtained in positive-ion mode; **C.** representative ion chromatograms of the three components of DCHD obtained in negative-ion mode


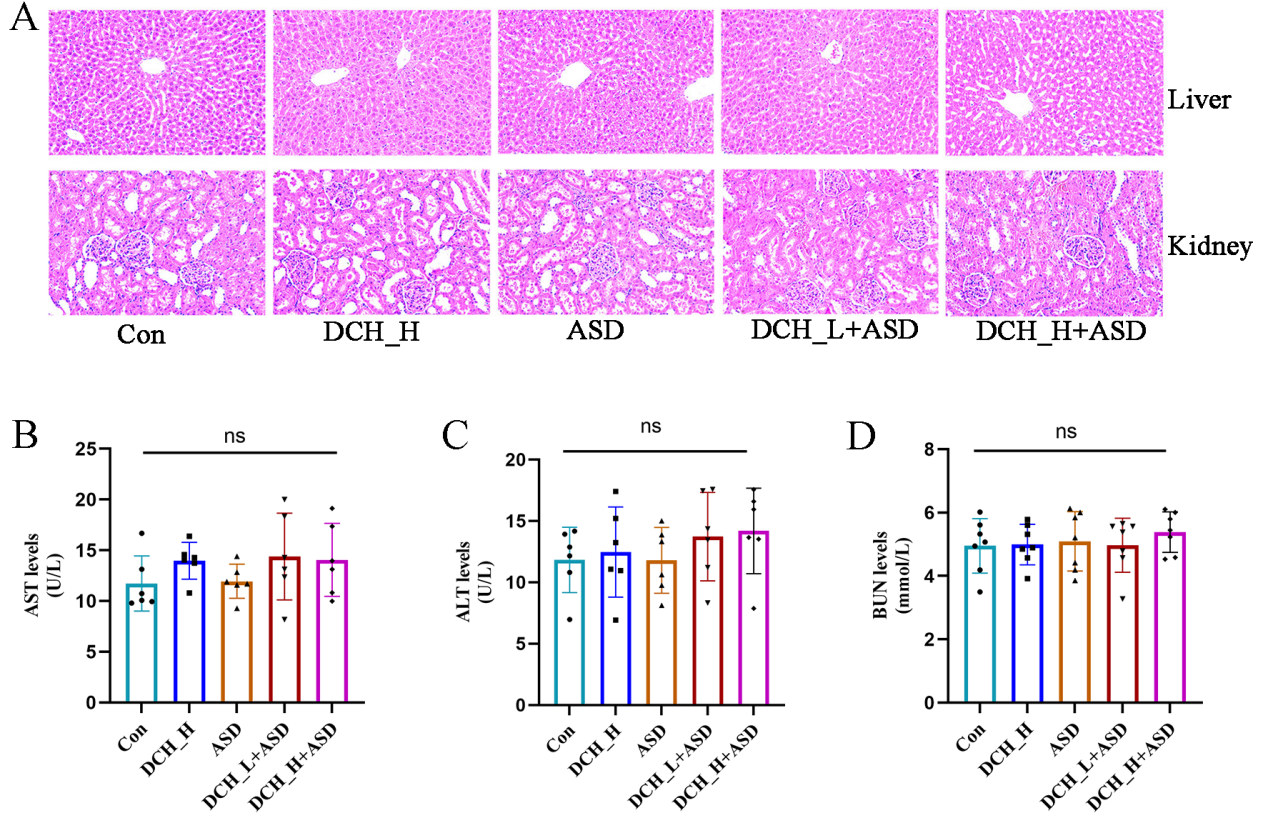


**Fig. S2 Effect of DCHD on liver histopathology in rats with PPA-induced autism.** **A**. Representative images of H&E staining of liver in rats following treatment with different DCHD doses by gavage; scale bars: 20 µm; **B**. serum AST level; **C**. serum ALT level; **D**. serum BUN level. AST, aspartate transaminase; ALT, alanine transaminase; BUN, blood urea nitrogen. Data are plotted as ± standard error of the mean. One-way ANOVA followed by Tukey’s post hoc test was used for multiple pairwise comparison. **p* < 0.05, ***p* < 0.01, ****p* < 0.001, ns, not significant.


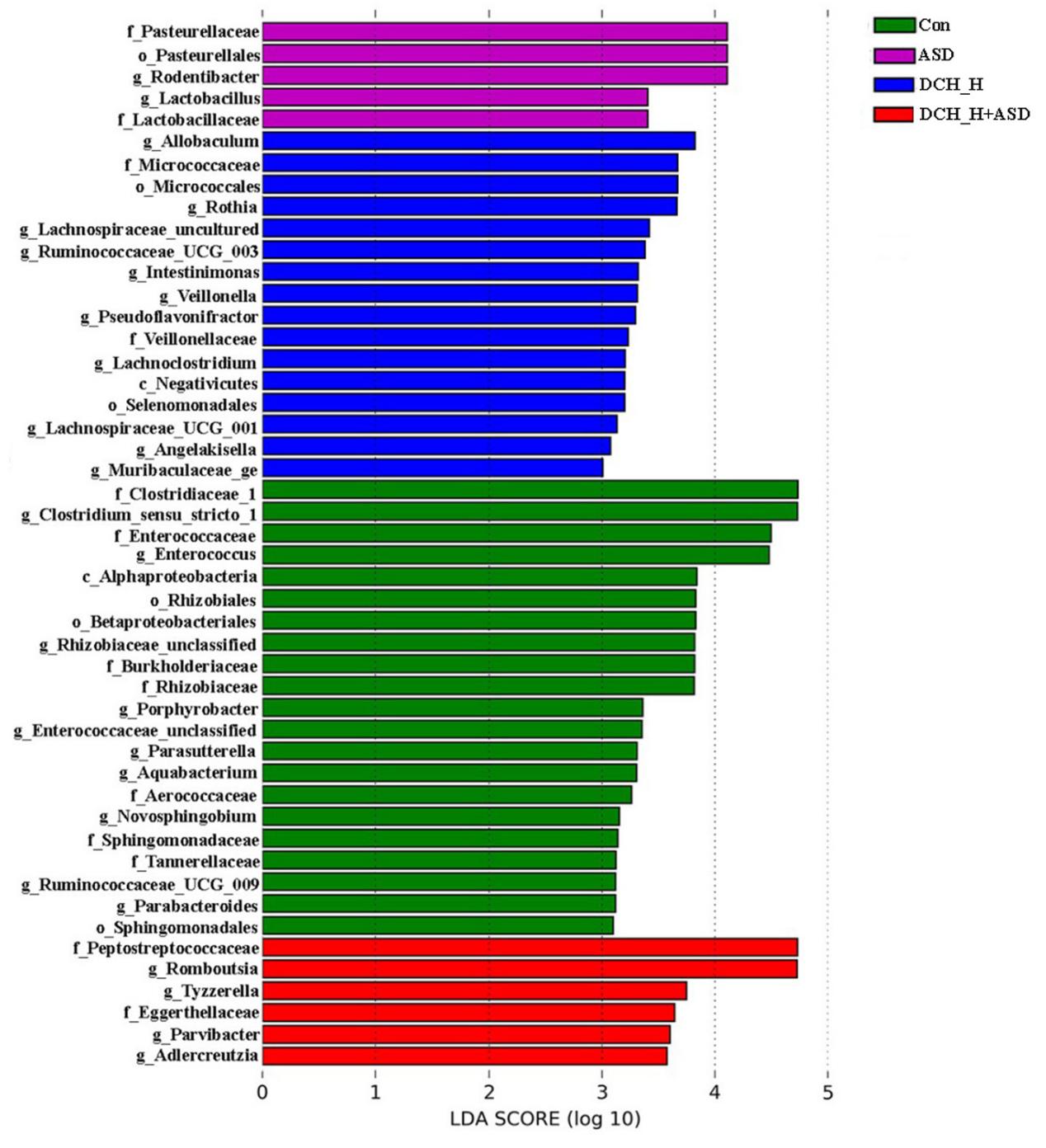


**Fig. S3. Cladogram of the LDA analysis of the gut microbiota composition in different groups.**


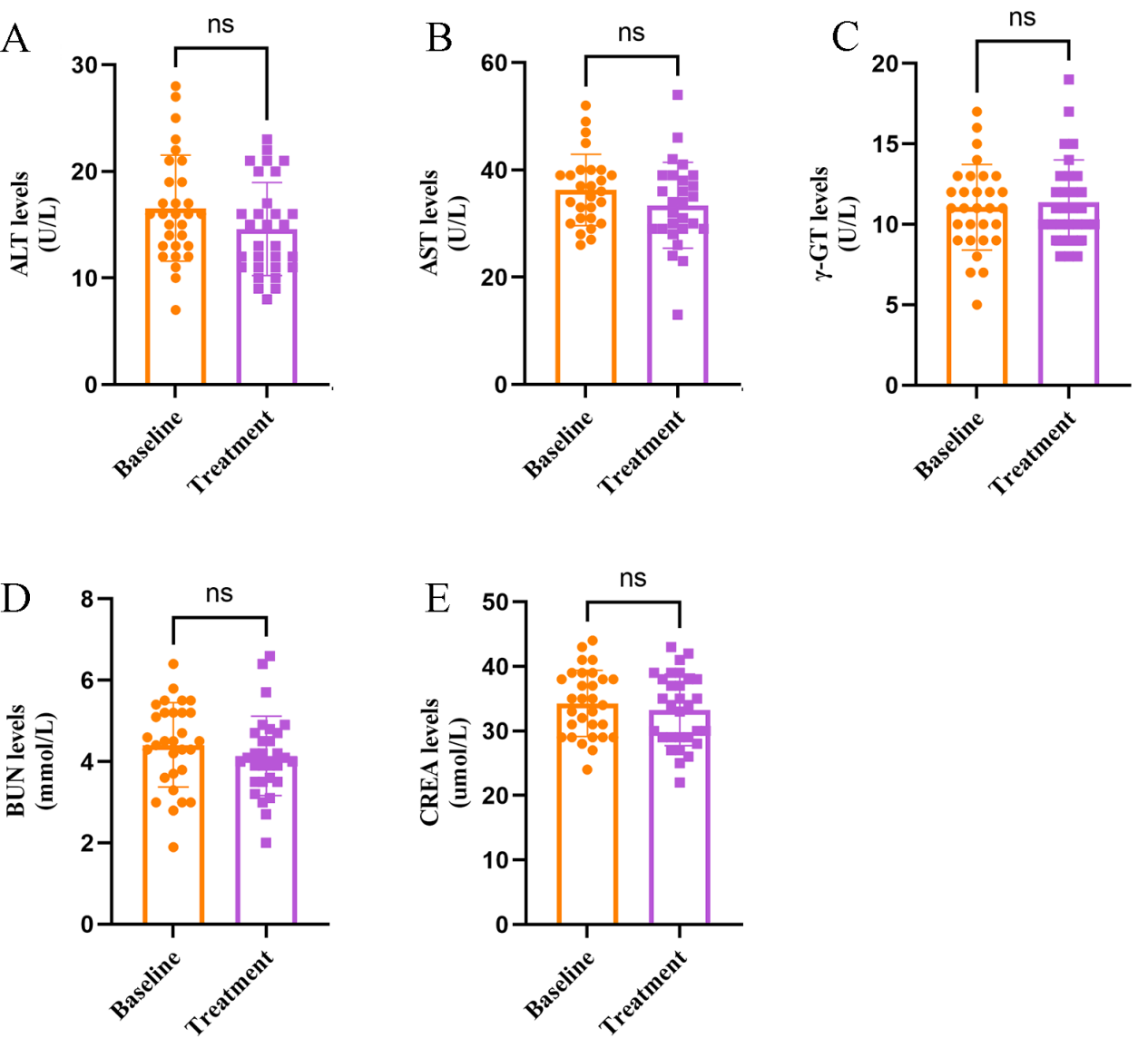


**Fig. S4 Effect of *Dachaihu* decoction on liver function and kidney function in children with ASD. A-E.** Serum levels of ALT, AST, γ-GT, BUN and CREA.One-way ANOVA followed by Tukey’s post hoc test was used for multiple pairwise comparison. ns, not significant.
